# Hsa_circ_0000199 facilitates chemo-tolerance of triple-negative breast cancer by interfering with miR-206/613-led PI3K/Akt/mTOR signaling

**DOI:** 10.18632/aging.202415

**Published:** 2021-01-20

**Authors:** Hongchang Li, Wen Xu, Zhihua Xia, Weiyan Liu, Gaofeng Pan, Junbin Ding, Jindong Li, Jianfa Wang, Xiaofeng Xie, Daowen Jiang

**Affiliations:** 1 Department of General Surgery, Institute of Fudan-Minhang Academic Health System, Minhang Hospital, Fudan University, Shanghai 201100, China; 2 State Key Laboratory of Bioreactor Engineering and Shanghai Key Laboratory of New Drug Design, School of Pharmacy, East China University of Science and Technology, Shanghai 200237, China; 3 Department of General Surgery, Putuo Hospital, Shanghai University of Traditional Chinese Medicine, Shanghai 200062, China; 4 Department of General Surgery, Shuguang Hospital Affiliated to Shanghai University of Traditional Chinese Medicine, Shanghai 200021, China

**Keywords:** hsa_circ_0000199, triple-negative breast cancer, miR-613, miR-206, PI3K/Akt/mTOR signaling

## Abstract

Increasing attentions have been paid to the role of circRNAs in the etiology of triple-negative breast cancer (TNBC), and we strived to figure out the association of circRNA AKT3/miRNA axis with TNBC chemo-resistance. Altogether 207 BC patients were divided into TNBC group (n=83) and non-TNBC group (n=124), and MCF-10A, MDA-MB-231, MDA-MB-468, SK-BR-3 and MCF-7 cell lines were prepared in advance. Expressions of AKT3-derived circRNAs and relevant miRNAs in the TNBC tissues and cell lines were determined by employing real-time polymerase chain reaction (PCR). It was indicated that hsa_circ_0000199 expression was higher in TNBC tissues than in non-TNBC tissues, and high hsa_circ_0000199 expression was predictive of large tumor size, advanced TNM grade, high Ki-67 level and poor 3-year survival of TNBC patients (all *P*<0.05). Furthermore, miR-613 and miR-206 were sponged and negatively regulated by hsa_circ_0000199 (*P*<0.001), and PI3K/Akt/mTOR signaling was depressed by si-hsa_circ_0000199 in TNBC cell lines (*P*<0.01). Ultimately, miR-206/miR-613 inhibitor reversed impacts of si-hsa_circ_0000199 on PI3K/Akt/mTOR signaling, proliferation, migration, invasion, chemo-sensitivity and autophagy of TNBC cells (all *P*<0.01). Conclusively, silencing of hsa_circ_0000199 enhanced TNBC chemo-sensitivity by promoting miR-206/miR-613 expression and deactivating PI3K/Akt/mTOR signaling, which was conducive to improving chemotherapeutic efficacy of TNBC patients.

## INTRODUCTION

Triple-negative breast cancer (TNBC), making up 10%~20% of breast cancer (BC) cases [1], revealed clinical attributes of malignant invasion, aggressive lymph-node metastasis and high recurrence [2]. Owing to shortages of estrogen receptor (ER), progesterone receptor (PR) and human epidermal growth factor receptor 2 (Her-2), endocrino- and trastuzumab-based therapies that worked for other BC subtypes were no longer suitable for TNBC treatment [3]. Instead, chemotherapies have been broadly applied to benefit TNBC patients, yet drug-tolerance rendered these strategies less efficacious than anticipated [4], which underscored the necessity of elucidating molecular mechanisms implicit in TNBC chemo-resistance.

Circular RNAs (circRNAs), originally mistaken as non-function products of RNA splicing, held potential to diagnose malignancies, and their dysfunction could powerfully drive progression of tumors [5], including BC [6], gastric cancer [7], glioma [8] and colorectal cancer [9]. Specifically, high expression of circKIF4A was associated with elevated likelihood of TNBC onset [10], and survival of TNBC patients was prolonged when they carried low expression of circAGFG1 [11]. Furthermore, knockout of circGFRA1 dampened proliferation and enabled apoptosis of TNBC cells [12], while adriamycin (ADM)-sensitivity of MCF7 cell line was encouraged after silencing of hsa_circ 0006528 [13]. Despite the growing recognition that circRNAs mattered in BC etiology, few circRNA-centric signaling pathways were verified to account for intensified drug-resistance in TNBC.

Intriguingly, circRNAs which were derived from tumor-activating/deactivating genes played similar roles in carcinogenesis, such as hsa_circ_0000543 (gene symbol: DAAM1) and hsa_circ_0000285 (gene symbol: HIPK3) [14–17]. Built on this assumption, circRNAs produced from AKT3, an oncogene in melanoma [18], hepatocellular carcinoma [PMID: 25370363] and TNBC [19], were also likely to be responsible for tumor progression, including TNBC. Furthermore, apart from spurring proliferation, migration and invasion of cancer cells [20], AKT3 also enabled rising tamoxifen-resistance in ErbB2(+) BC cells [21]. However, whether AKT3-derived circRNAs also enhanced chemo-resistance and promoted deterioration of neoplasms (e.g. TNBC) remained ambiguous. In addition, circRNAs were expected to facilitate carcinogenesis by sponging cancer-specific miRNAs and then stimulating translation of oncogenes [22]. For example, hsa_circRNA_002178 was reported to foster migration and invasion of BC cells by sponging miR-328-3p and motivating COL1A1 expression [23]. Nonetheless, so far few researches were able to surface the association of circRNA AKT3/miRNA network with TNBC progression and chemo-tolerance.

To bridge this gap, this investigation was intended to elucidate the contribution of circRNA AKT3-centric miRNA axes underlying TNBC etiology, which might help to address concerns over TNBC chemo-resistance.

## RESULTS

### Association of hsa_circ_0000199 expression with clinico-pathological features of TNBC patients

**Figure 1:**
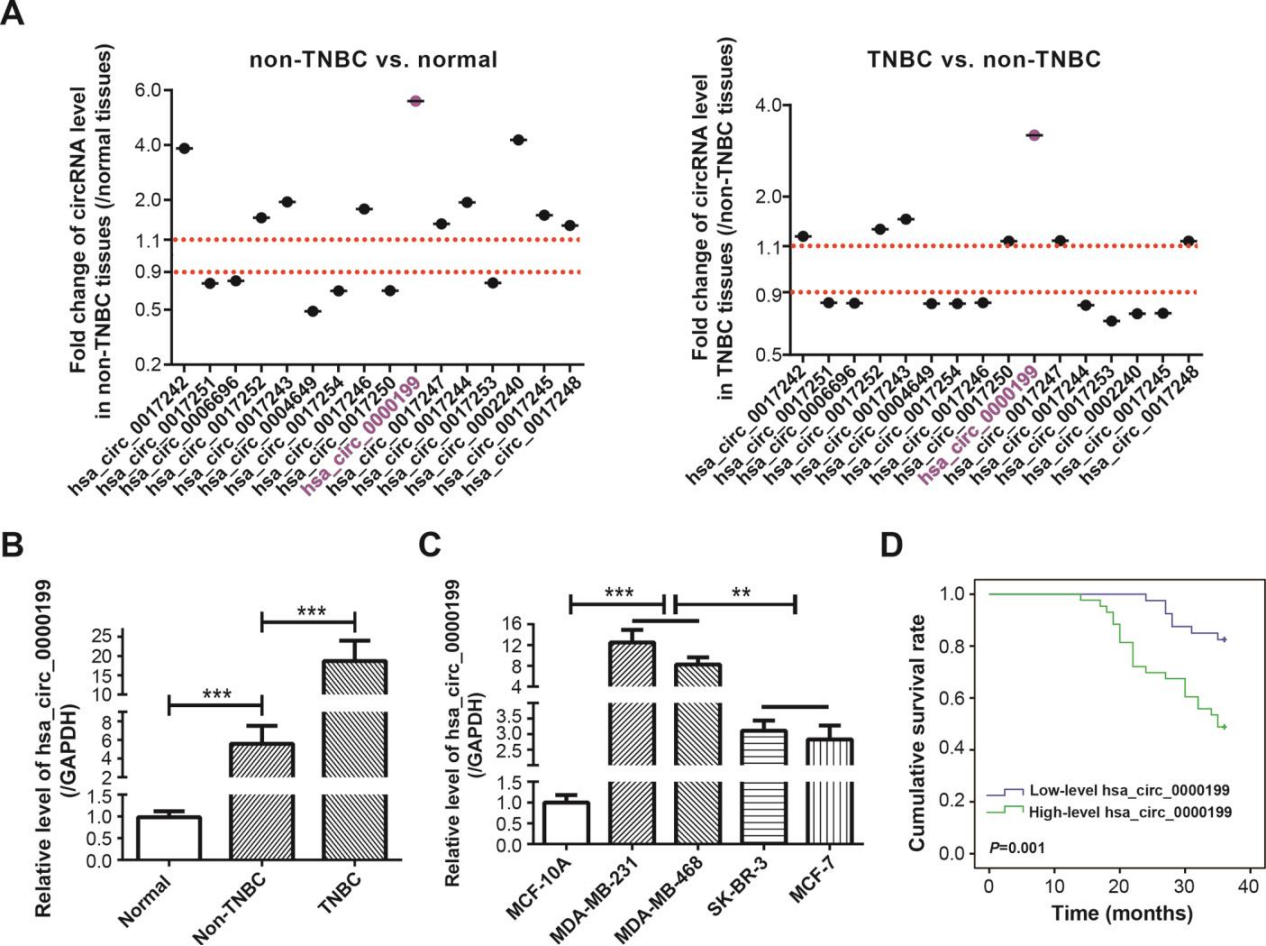
**Clinical value of circRNA AKT3 in triple-negative breast cancer (TNBC).** (**A**) Fold change of AKT3-derived circRNAs were determined in BC tissues of other subtypes (i.e. non-TNBC) as relative to normal tissues, and also in TNBC tissues as relative to BC tissues of other subtypes. (**B**) Hsa_circ_0000199 expression was compared among normal tissues, TNBC tissues and BC tissues of other subtypes. ***: *P*<0.001. (**C**) Hsa_circ_0000199 expression was measured among MCF-10A, MDA-MB-231, MDA-MB-468, SK-BR-3 and MCF-7 cell lines. **: *P*<0.01; ***: *P*<0.001. (**D**) Hsa_circ_0000199 expression was associated with 3-year survival of TNBC patients.

There were altogether 16 circRNAs retrievable from both ENCORI website (http://starbase.sysu.edu.cn/) [24] and CircInteractome website (https://circinteractome.nia.nih.gov/) [25], including hsa_circ_0017242, hsa_circ_0017251, hsa_circ_0006696, hsa_circ_0017252, hsa_circ_0017243, hsa_circ_0004649, hsa_circ_0017254, hsa_circ_0017246, hsa_circ_0017250, hsa_circ_0000199, hsa_circ_0017247, hsa_circ_0017244, hsa_circ_0017253, hsa_circ_0002240, hsa_circ_0017245 and hsa_circ_0017248 (Supplementary Table 3), and miRNAs potentially sponged by the circRNAs were included in Supplementary Table 4. Expressions of the circRNAs were tentatively compared among adjacent normal tissues (n=60), TNBC tissues (n=30) and non-TNBC tissues (n=30) (Figure 1A). It was demonstrated that hsa_circ_0000199 expression in TNBC tissues was increased to 3.34 times of that in non-TNBC tissues (*P*<0.001), and hsa_circ_0000199 expression in non-TNBC tissues reached 5.60 folds of that in adjacent normal tissues (*P*<0.001). Hsa_circ_0017242, has_circ_0017243, hsa_circ_0017246, hsa_circ_0017244 and hsa_circ_0017240 were also up-regulated in non-TNBC tissues in comparison to adjacent normal tissues (all *P*<0.001), however, their expressional change between TNBC and non-TNBC tissues were not so pronounced as hsa_circ_0000199.

To emphasize the part of hsa_circ_0000199 in TNBC, a larger crowd of TNBC patients (n=83) and non-TNBC patients (n=124) were incorporated, which revealed that hsa_circ_0000199 expression was indeed higher in TNBC tissues than in non-TNBC tissues (*P*<0.0001) (Figure 1B). Consistently, TNBC cell lines (i.e. MDA-MB-231 and MDA-MB-468) also expressed larger amounts of hsa_circ_0000199 than normal breast epithelial cell line (i.e. MCF-10A) and BC cell lines of other subtypes (i.e. SK-BR-3 and MCF-7) (*P*<0.001) (Figure 1C). Furthermore, TNBC patients and non-TNBC patients were separately sub-grouped based on their median expression of hsa_circ_0000199 (TNBC: 5.36; non-TNBC: 1.43) (Table 1). It was indicated that highly-expressed (>5.36) hsa_circ_0000199 was associated with large tumor size (diameter > 5 cm) (*P*=0.010), advanced TNM grade (G3) (*P*=0.003) and high Ki-67 proportion (>14%) (*P*=0.012) of TNBC patients, while hardly any statistical significance was discernable among patients of non-TNBC group (all *P*>0.05). Moreover, 3-year overall survival of TNBC patients was less desirable in the highly-expressed hsa_circ_0000199 group than in the lowly-expressed hsa_circ_0000199 group (*P*=0.001) (Figure 1D), and highly-expressed hsa_circ_0000199 (*P*=0.048), large tumor size (*P*=0.014) and high Ki-67 proportion (*P*=0.012) were independent predictors of unfavorable prognosis among the TNBC population (Table 2 and Supplementary Figure 1). All these results implied that hsa_circ_0000199 was a peculiar biomarker in estimating TNBC onset and prognosis among the Chinese cohort.

**Table 1:** Association of hsa_circ_0000199 expression with clinical features of breast cancer (BC) patients.

**Items**	**TNBC group (N=83)**						**Non-TNBC group (N=124)**					
	**High (N=43)**	**Low (N=40)**	**χ^2^**	***P***	**OR**	**95%CI**	**High (N=64)**	**Low (N=60)**	**χ^2^**	***P***	**OR**	**95%CI**
**Age (years old)**												
≤55	20	17					35	37				
>55	23	23	0.14	0.713	0.85	0.36-2.02	29	23	0.6195	0.431	1.33	0.65-2.73
**Diameter of lesion (cm)**												
≤5	27	35					52	44				
>5	16	5	6.70	0.010*	4.15	1.35-12.75	12	16	1.11	0.292	0.64	0.27-1.48
**Classification**												
IDC	39	31					41	46				
Others	4	9	2.73	0.098	0.35	0.10-1.26	23	14	2.35	0.125	1.84	0.84-4.05
**TNM grade**												
G1+G2	29	37					54	53				
G3	14	3	8.67	0.003*	6.40	1.67-24.48	10	7	0.4102	0.522	1.40	0.50-3.96
**Ki-67**												
≤14%	15	25					43	38				
>14%	28	15	6.33	0.012*	3.11	1.27-7.62	21	22	0.203	0.652	0.84	0.40-1.77

BC: breast cancer; TNBC: triple-negative breast cancer; non-TNBC: BC patients who do not belong to TNBC subtype; High: high hsa_circ_0000199 expression; Low: low hsa_circ_0000199 expression; OR: odds ratio; CI: confidence interval; *: statistical significance.

**Table 2:** Association of clinical indicators with 3-year overall survival of triple-negative breast cancer (TNBC) patients.

**Items**	**Number of cases (n)**	**Uni-variate analysis**			**Multi-variate analysis**		
		**HR**	**95% CI**	***P* value**	**HR**	**95% CI**	***P* value**
**Age (years old)**							
≤50	37						
>50	46	0.593	0.285-1.232	0.161	0.672	0.313-1.445	0.309
**Diameter of lesion (cm)**							
≤5	62						
> 5	21	2.574	1.228-5.393	0.012*	2.696	1.222-5.946	0.014*
**Classification**							
IDC	70						
Others	13	1.227	0.468-3.218	0.677	2.354	0.795-6.973	0.122
**TNM grade**							
G1+G2	64						
G3	19	1.813	0.825-3.982	0.139	1.105	0.468-2.614	0.819
**Ki-67**							
≤14%	40						
>14%	43	3.218	1.423-7.279	0.005*	3.095	1.281-7.477	0.012*
**Relative level of hsa_circ_0000199**							
Low	40						
High	43	3.783	1.614-8.868	0.002*	2.91	1.008-8.401	0.048*

HR: hazard ratio; CI: confidence interval; *: statistical significance.

### Downstream miRNA network of AKT3-derived circRNAs in TNBC

**Figure 2:**
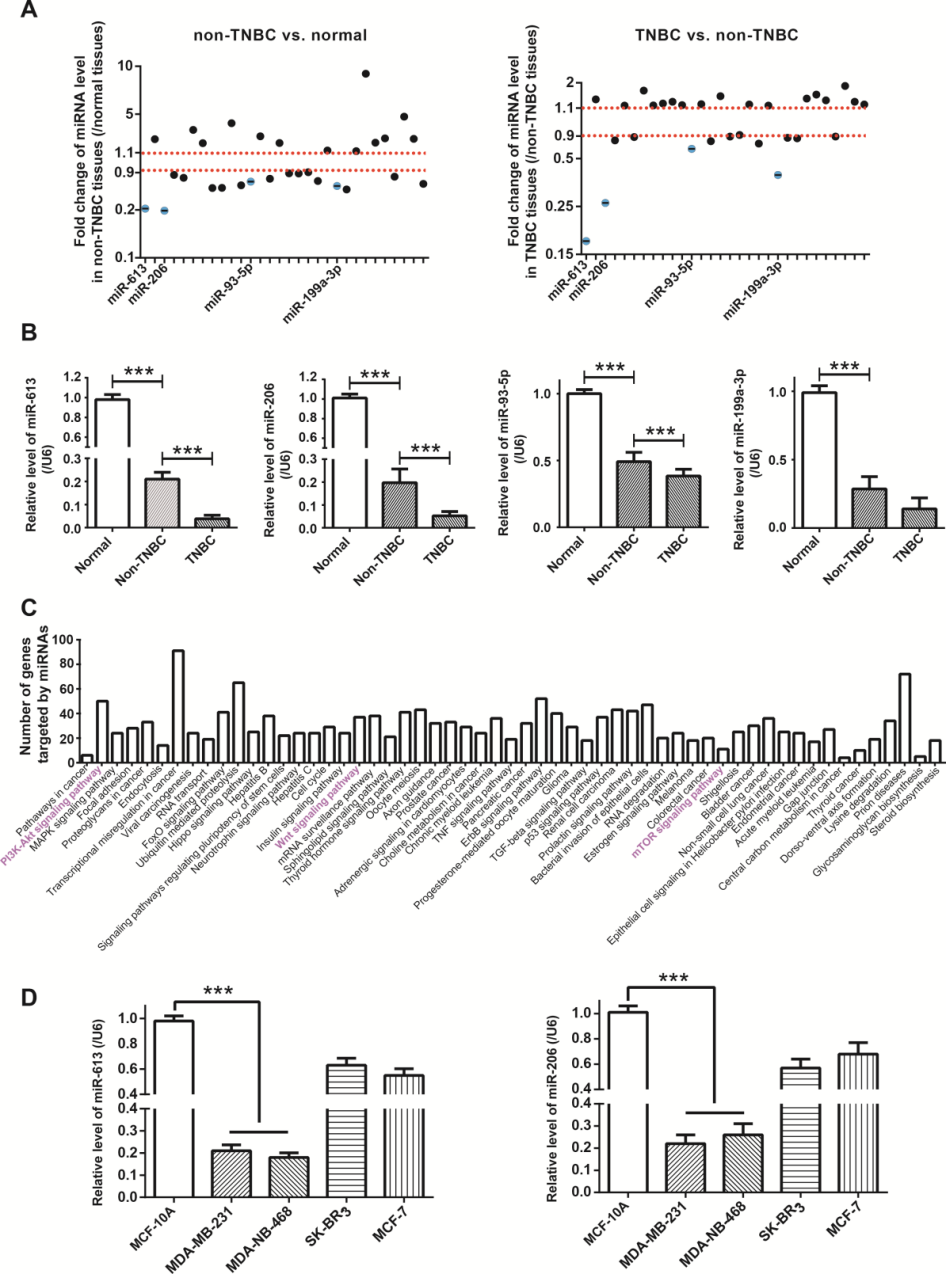
**Identification of miRNA network of circRNA AKT3 in triple-negative breast cancer (TNBC).** (**A**) Fold change of miRNAs, potentially sponged by significant AKT3-derived circRNAs, were determined in BC tissues of other subtypes (i.e. non-TNBC) as relative to normal tissues, as well as in TNBC tissues as relative to BC tissues of other subtypes. (**B**) Expressions of miR-613, miR-206, miR-93-5p and miR-199a-3p were determined in normal tissues, TNBC tissues and BC tissues of other subtypes (i.e. non-TNBC). ***: *P*<0.001. (**C**) KEGG pathways enriched by genes targeted by miR-613, miR-206, miR-93-5p and miR-199a-3p were drawn from miRPath online tool (http://snf-515788.vm.okeanos.grnet.gr/). (**D**) MiR-613 and miR-206 expressions were compared among MCF-10A, MDA-MB-231, MDA-MB-468, SK-BR-3 and MCF-7 cell lines. ***: *P*<0.001.

As concluded by KEGG database (https://www.kegg.jp/kegg/pathway.html), Notch signaling, Wnt-β actin signaling, PI3K/Akt/mTOR signaling and EGFR signaling were critical pathways inducing TNBC onset (https://www.kegg.jp/kegg-bin/highlight_pathway?scale=1.0&map=map05224&keyword=Triple%20negative%20breast%20cancer). It was noteworthy that a majority of miRNAs, which were potentially sponged by top 5 up-regulated circRNAs in BC (Figure 1A and Supplementary Table 4), were documented to intervene in the four signalings, and nine of them were pivotal indicators of TNBC progression (Supplementary Table 5)*.* After comparing expressions of the miRNAs among adjacent normal tissues (n=60), TNBC tissues (n=30) and non-TNBC tissues (n=30) (Figure 2A), we noticed that miR-613, miR-206, miR-93-5p and miR-199a-3p, which were down-regulated in non-TNBC tissues as relative to para-cancerous normal tissues (all *P*<0.001), exhibited lower expression in TNBC tissues than in non-TNBC tissues (all *P*<0.001) (Figure 2B). Furthermore, according to the estimation of miRPath online software (http://snf-515788.vm.okeanos.grnet.gr/) [26], genes targeted by the miR-613, miR-206, miR-93-5p and miR-199a-3p were enriched in mTOR signaling and PI3K/Akt signaling (Figure 2C), which insinuated that the miRNAs were probably implicated in TNBC pathogenesis by dysregulating PI3K/Akt/mTOR signaling.

### Hsa_circ_0000199 sponged miR-613/miR-206 and down-regulated their expression in TNBC cell lines

**Figure 3:**
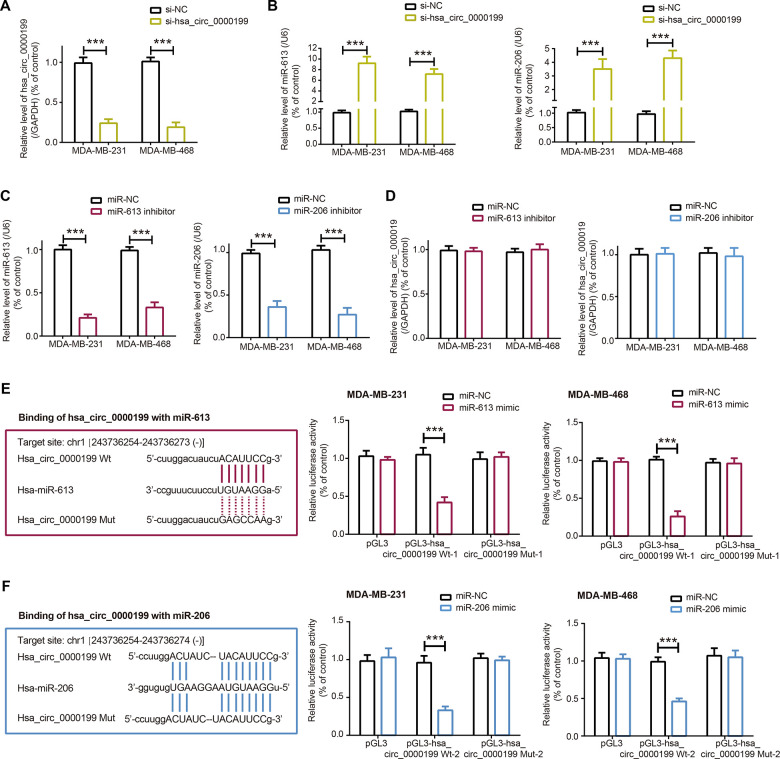
**Sponged relationship between hsa_circ_0000199 and miR-613/miR-206 in triple-negative breast cancer (TNBC).** (**A**) Hsa_circ_0000199 expression in MDA-MB-231 and MDA-MB-468 cell lines was determined after transfection of si-hsa_circ_0000199. ***: *P*<0.001. (**B**) MiR-613 and miR-206 expressions in MDA-MB-231 and MDA-MB-468 cell lines were compared between si-NC group and si-hsa_circ_0000199 group. ***: *P*<0.001. (**C**) MiR-613 and miR-206 expressions were assessed after transfection of their respective inhibitors into MDA-MB-231 and MDA-MB-468 cell lines. ***: *P*<0.001. (**D**) Hsa_circ_0000199 expression in MDA-MB-231 and MDA-MB-468 cell lines was evaluated after transfection of miR-613/miR-206 inhibitor. (**E**) Hsa_circ_0000199 sponged miR-613 in certain sites, and luciferase activity of MDA-MB-231 and MDA-MB-468 cell lines was compared between pGL3-hsa_circ_0000199 Wt+miR-613 mimic group and pGL3-hsa_circ_0000199 Wt+miR-NC group. ***: *P*<0.001. (**F**) MiR-206 was sponged by hsa_circ_0000199, and luciferase activity of MDA-MB-231 and MDA-MB-468 cell lines was determined after co-transfection of pGL3-hsa_circ_0000199 Wt and miR-206 mimic/miR-NC. ***: *P*<0.001.

When compared with SK-BR-3 and MCF-7 cell lines, expressions of miR-613 and miR-206 were dramatically reduced in TNBC cell lines (i.e. MDA-MB-231 and MDA-MB-468) (*P*<0.001) (Figure 2D), which further corroborated that the couple of miRNAs were specific protectors for TNBC [16, 27]. After transfection of si-hsa_circ_0000199, hsa_circ_0000199 expression fell significantly in MDA-MB-231 and MDA-MB-468 cell lines (*P*<0.001) (Figure 3A), and expressions of miR-613 and miR-206 were marked increased (*P*<0.001) (Figure 3B). Nevertheless, miR-613/miR-206 inhibitor, which decreased miR-613/miR-206 expression in TNBC cell lines (*P*<0.001) (Figure 3C), appeared incapable of altering hsa_circ_0000199 expression in MDA-MB-231 and MDA-MB-468 cell lines (all *P*>0.05) (Figure 3D).

In addition, luciferase activity of TNBC cells was weakened after combined transfection of miR-613/miR-206 mimic and pGL3-hsa_circ_0000199, when compared with TNBC cells of miR-613/miR-206 mimic+hsa_circ_0000199 Wt group and hsa_circ_0000199 Mut+miR-NC group (*P*<0.001) (Figure 3E, 3F). Meanwhile, there was no statistical difference in luciferase activity of TNBC cells between miR-613/miR-206 mimic+hsa_circ_0000199 Wt group and hsa_circ_0000199 Mut+miR-NC group (*P*>0.05). Collectively, it was implied that miR-613 and miR-206 were sponged and down-regulated by hsa_circ_0000199 in TNBC.

### MiR-613 and miR-206 hindered impacts of hsa_circ_0000199 on PI3K/Akt/mTOR signaling

**Figure 4:**
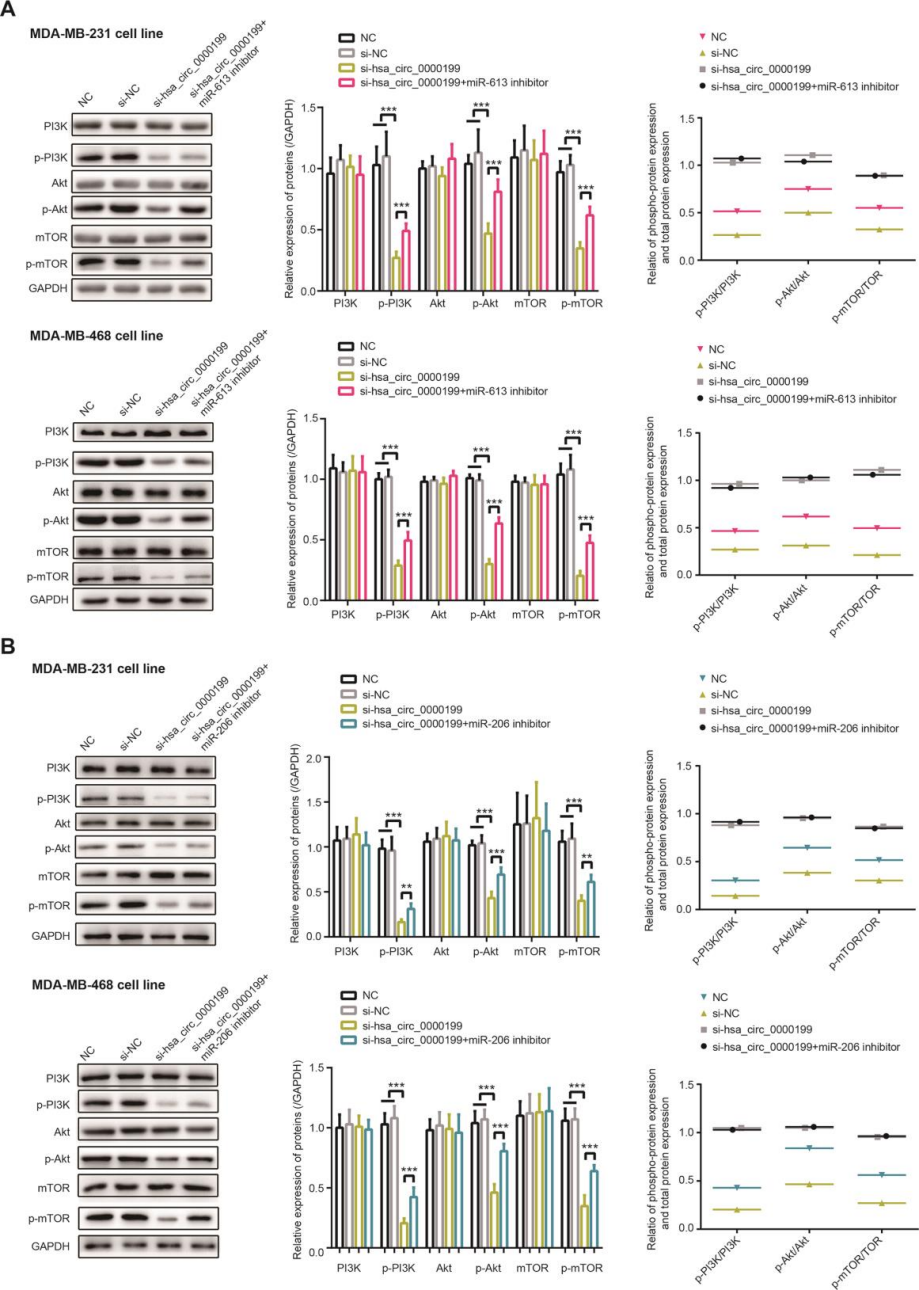
**PI3K/AKT/mTOR signaling was modified by hsa_circ_0000199-miR-613/miR-206 axis in triple-negative breast cancer (TNBC).** (**A**) Protein levels of PI3K, p-PI3K, AKT, p-AKT, mTOR and p-mTOR, as well as ratios of PI3K/p-PI3K, AKT/p-AKT and mTOR/p-mTOR, were compared among NC, si-NC, si-hsa_circ_0000199+miR-613 inhibitor and si-hsa_circ_0000199 groups. ***: *P*<0.001. (**B**) Protein levels of PI3K, p-PI3K, AKT, p-AKT, mTOR and p-mTOR, as well as ratios of PI3K/p-PI3K, AKT/p-AKT and mTOR/p-mTOR in MDA-MB-231 and MDA-MB-468 cell lines, were determined after treatments of NC, si-NC, si-hsa_circ_0000199+miR-206 inhibitor and si-hsa_circ_0000199. **: *P*<0.01; ***: *P*<0.001.

Phosphorylation of PI3K, Akt and mTOR was depressed in MDA-MB-231 and MDA-MB-468 cell lines, after silencing of hsa_circ_0000199 (all *P*<0.001) (Figure 4). Powered by si-hsa_circ_0000199, p-PI3K/PI3K ratio, p-Akt/Akt ratio and p-mTOR/mTOR ratio were also decreased in the TNBC cells, when compared with NC group and si-NC group. Nevertheless, under co-transfection of si-hsa_circ_0000199 and miR-206/miR-613 inhibitor, phosphorylation of PI3K, Akt and mTOR were improved in MDA-MB-231 (all *P*<0.01) and MDA-MB-468 (all *P*<0.001) cell lines, in comparison to si-hsa_circ_0000199 transfection alone. Moreover, p-PI3K/PI3K ratio, p-Akt/Akt ratio and p-mTOR/mTOR ratio were raised in the si-hsa_circ_0000199+miR-206/miR-613 inhibitor group as relative to si-hsa_circ_0000199 group. Given that PI3K/Akt/mTOR signaling dampened cell autophagy [28], which exerted dual impacts on neoplastic chemo-resistance [29, 30], hsa_circ_0000199-centric miR-206/miR-613 axes might also be responsible for chemo-tolerance and disordered cell autophagy in TNBC.

### MiR-206 and miR-613 undermined contribution of hsa_circ_0000199 to proliferation, migration, invasion and chemo-sensitivity of TNBC cells

**Figure 5:**
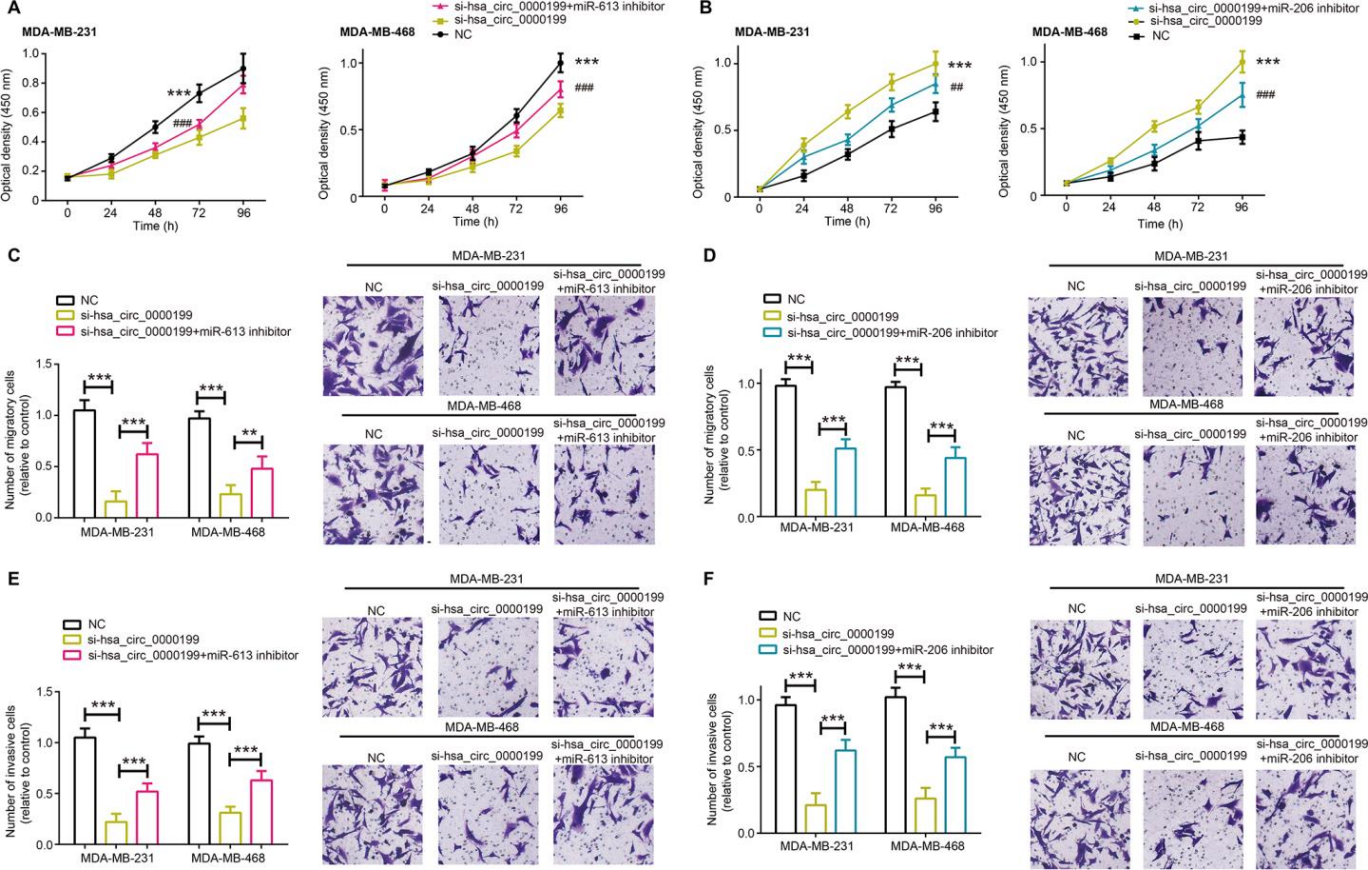
**MiR-613 and miR-206 were involved in modulating contribution of hsa_circ_0000199 to activity of triple-negative breast cancer (TNBC) cells.** (**A**, **B**) Proliferation of MDA-MB-231 and MDA-MB-468 cell lines were compared after treatments of si-hsa_circ_0000199+miR-613/miR-206 inhibitor, si-hsa_circ_0000199 and none. *: compared with NC group; #: compared with si-hsa_circ_0000199 group; **/##: *P*<0.01; ***/###: *P*<0.001. (**C**, **D**) Migration of MDA-MB-231 and MDA-MB-468 cell lines were appraised among si-hsa_circ_0000199+miR-613/miR-206 inhibitor, si-hsa_circ_0000199 and NC groups. **: *P*<0.01; ***: *P*<0.001. (**E**, **F**) The invasive capability of MDA-MB-231 and MDA-MB-468 cell lines were evaluated after treatments of si-hsa_circ_0000199+miR-613/miR-206 inhibitor, si-hsa_circ_0000199 and none. ***: *P*<0.001.

Malignant activities of MDA-MB-231 and MDA-MB-468 cell lines were all decelerated after silencing of hsa_circ_0000199, regardless of proliferation (Figure 5A, 5B, *P*<0.001), migration (Figure 5C, 5D, *P*<0.001) or invasion (Figure 5E, 5F, *P*<0.001). Nonetheless, miR-613 inhibitor and miR-206 inhibitor abated the suppressive impact of si-hsa_circ_0000199 on proliferation (Figure 5A, 5B), migration (Figure 5C, 5D) and invasion (Figure 5E, 5F) of MDA-MB-231 and MDA-MB-468 cell lines, specifically embodied as that proliferation, migration and invasion of TNBC cells were encouraged in si-hsa_circ_0000199+miR-206/miR-613 inhibitor group as relative to si-hsa_circ_0000199 group (all *P*<0.01).

**Figure 6:**
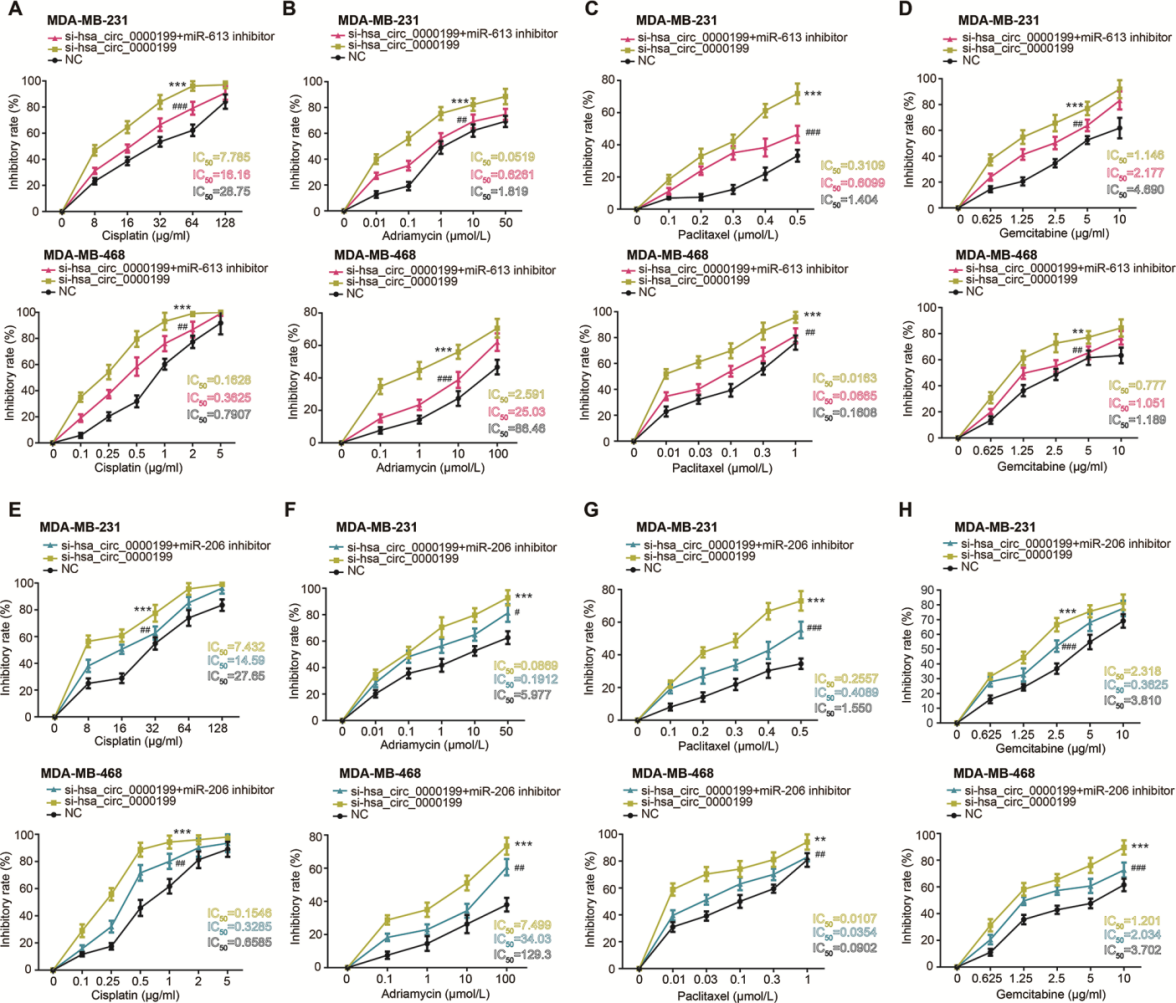
**Hsa_circ_0000199-miR-613/miR-206 axis was implicated in modifying chemosensitivity of triple-negative breast cancer (TNBC) cells.** (**A**–**D**) Resistance of MDA-MB-231 and MDA-MB-468 cell lines against cisplatin (**A**), adriamycin (**B**), paclitaxel (**C**) and gemcitabine (**D**) was compared among si-hsa_circ_0000199+miR-613 inhibitor, si-hsa_circ_0000199 and NC groups. *: compared with NC group; #: compared with si-hsa_circ_0000199 group; **/##: *P*<0.01; ***/###: *P*<0.001. (**E**–**H**) Sensitivity of MDA-MB-231 and MDA-MB-468 cell lines responding to cisplatin (**E**), adriamycin (**F**), paclitaxel (**G**) and gemcitabine (**H**) was assessed after treatments of si-hsa_circ_0000199+miR-206 inhibitor, si-hsa_circ_0000199 and none. *: compared with NC group; #: compared with si-hsa_circ_0000199 group; */#: *P*<0.05; **/##: *P*<0.01; ***/###: *P*<0.001.

Furthermore, MDA-MB-231 and MDA-MB-468 cell lines in the si-hsa_circ_0000199 group became vulnerable to the killing effect of cisplatin, adriamycin, paclitaxel and gemcitabine, when compared with NC group and si-NC group (all *P*<0.001) (Figure 6). However, tolerances of TNBC cells against cisplatin, adriamycin, paclitaxel and gemcitabine were strengthened in the si-hsa_circ_0000199+miR-613/miR-206 inhibitor group in comparison to si-circ_0000199 group (all *P*<0.01) (Figure 6).

### MiR-206 and miR-613 disturbed influence of hsa_circ_0000199 on TNBC autophagy

**Figure 7:**
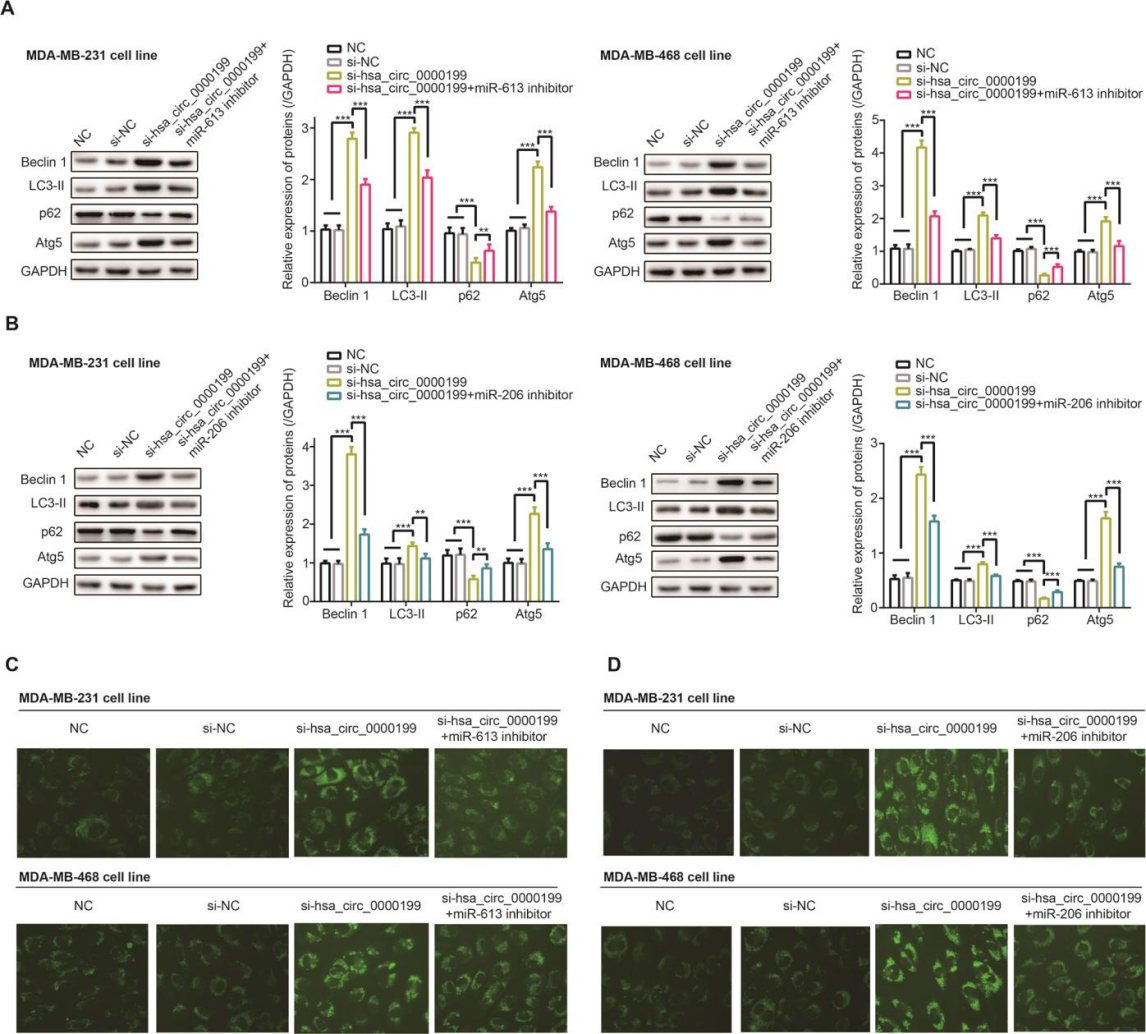
**Hsa_circ_0000199-miR-613/miR-206 axis participated in regulating autophagy of triple-negative breast cancer (TNBC) cells.** (**A**) Beclin-1, LC3-II, p62 and Atg5 expressions were determined in MDA-MB-231 and MDA-MB-468 cell lines treated by NC, si-NC, si-hsa_circ_0000199 and si-hsa_circ_0000199+miR-206 inhibitor. **: *P*<0.01; ***: *P*<0.001. (**B**) Beclin-1, LC3-II, p62 and Atg5 expressions were detected among MDA-MB-231 and MDA-MB-468 cell lines of NC, si-NC, si-hsa_circ_0000199 and si-hsa_circ_0000199+miR-613 inhibitor groups. **: *P*<0.01; ***: *P*<0.001. (**C**) Monodansylcadaverine (MDC) fluorescence intensity was monitored among MDA-MB-231 and MDA-MB-468 cell lines transfected by NC, si-NC, si-hsa_circ_0000199 and si-hsa_circ_0000199+miR-613 inhibitor. (**D**) MDC fluorescence intensity of MDA-MB-231 and MDA-MB-468 cell lines was determined among NC, si-NC, si-hsa_circ_0000199 and si-hsa_circ_0000199+miR-206 inhibitor groups.

Beclin1 and LC3-II were a couple of proteins indispensable to cell autophagy [31, 32], and p62 was degraded in case of cell autophagy [33]. Not only that, Atg5 expression was promoted during autophagy, and its combination with Atg12 could drive extension of autophagosome membrane [34]. Here we observed that Beclin1, LC3-II and p62 expressions were evidently boosted, and p62 expression was down-regulated in MDA-MB-231 and MDA-MB-468 cell lines after transfection of si-has_circ_0000199 (*P*<0.001) (Figure 7A, 7B). Consistently, MDC-positive particles in the form of bright blue dots were abundantly present in MDA-MB-231 and MDA-MB-468 cell lines of si-hsa_circ_0000199 group in comparison to si-NC group, and the number of MDC-positive particle was falling in si-hsa_circ_0000199+3-MA group as relative to si-hsa_circ_0000199 group (Supplementary Figure 2).

Nonetheless, protein levels of beclin1, LC3-II and Atg5 were down-regulated, accompanied by up-regulated expression of p62, in MDA-MB-231 and MDA-MB-468 cell lines of si-hsa_circ_0000199+miR-206/miR-613 inhibitor group in comparison to TNBC cells of si-hsa_circ_0000199 group (all *P*<0.01) (Figure 7A, 7B). Co-transfection of si-hsa_circ_0000199 and miR-206/miR-613 inhibitor engendered less MDA-positive particles than simply transfection of si-circ_0000199 (Figure 7C).

## DISCUSSION

Despite with a 5-year survival of 90%, there were up to 41,760 American females dying of BC in 2019, covering 15% of all tumor deaths [35]. TNBC, a BC subtype notorious for high odds of recurrence and metastasis [36], was managed principally by various chemotherapies, whose efficacy, however, was reduced owing to drug-resistance. Therefore, clarification of TNBC etiology was urgently required, and growing interests were sparked concerning the implication of circRNA-led miRNA network in TNBC.

Distinct from linear RNAs with the structure of 5’-cap and 3’-tail, circRNAs in the shape of closed rings were produced through back-splicing approach [37], which made it tough to degrade circRNA with exonuclease and thereby maintained circRNA stability. Thanks to this trait, circRNAs were capable of reflecting cancer progression sensitively, including bladder cancer [38], hepatocellular cancer [39], laryngeal cancer [40] and basal cell carcinoma [41]. With regard to BC, hsa_circ_006054 combined with hsa_circ_100219 and has_circ_406697 excelled in diagnosing BC patients from healthy volunteers [42], and molecular results showed that proliferation of MDA-MB-231 cell line was boosted by circDENND4C in the oxygen-free context [43]. Nonetheless, circRNAs available to differentiate BC subtypes (e.g. TNBC) were poorly known, let alone their sophisticated function in TNBC etiology [12, 44]. In this investigation, AKT3-derived hsa_circ_0000199 was found to specifically over-express in TNBC (Figure 1), and its high expression was associated with clinical symptoms of TNBC patients, rather than the whole BC population (Tables 1, 2). However, whether this result could be generalized to other populations demanded more researches.

The competing endogenous RNA (ceRNA) hypothesis introduced that circRNAs could sponge miRNAs with their miRNA response elements (MREs), and then lessened impacts of miRNAs on neoplastic development [45]. Taking BC for instance, circRNA antisense to the cerebellar degeneration-related protein 1 transcript (CDR1-AS) sponged miR-7 and reduced its expression, thereby slowing down BC exacerbation [46]. In agreement with this theory, miR-613 and miR-206 were negatively modified by hsa_circ_0000199 in TNBC cells after being targeted by it (Figures 2, 3), and they also participated in the contribution of hsa_circ_0000199 to malignant activities of TNBC cells (Figures 5–7). Collectively, si-hsa_circ_0000199 might curb worsening of TNBC through suppressing anti-oncogenic functions of miR-613 and miR-206, which have been extensively published. To be specific, miR-206 restrained multiplication of MCF-7 cell line [47] and metastasis of MDA-MB-231 cell line [48], and miR-613 antagonized progression of gastric cancer [49], bladder cancer [50], osteosarcoma [51], thyroid papillary carcinoma [52] and TNBC [16]. However, there was a contradictory finding which stated that miR-613 deteriorated colon cancer by targeting ATOH1 and motivating JNK1 signaling [53]. We speculated that different cell types used and distinct experimental procedures followed could account for this paradox, yet convincing evidence was entailed.

Additionally, PI3K/Akt/mTOR signaling, downstream pathway of hsa_circ_0000199-miR-206/miR-613 axis in TNBC (Figure 4), was reported to stimulate tumor onset and to potentiate metastasis and proliferation of tumor (e.g. TNBC) cells [54]. Moreover, phosphorylated mTOR was measurable in a larger share of TNBC patients than in non-TNBC patients, which stressed that PI3K/Akt/mTOR signaling could matter in TNBC as compared with other BC subtypes [55]. Furthermore, rapamycin treatment, a common inhibitor of mTOR signaling, not merely strengthened anti-TNBC power of adriamycin in nude mice [56], but also improved paclitaxel’s performance in fighting against TNBC [57], implying that it was practicable to raise TNBC chemo-sensitivity by attenuating PI3K/Akt/mTOR signaling. In addition, autophagy, which served bi-directional roles in tumors [58], was induced when PI3K/Akt/mTOR signaling was obstructed [28]. This physiological change was likely to weaken tumor development by facilitating apoptosis of tumor cells, which also explained increased chemo-sensitivity of BC [59]. Allowing for multiple roles performed by PI3K/Akt/mTOR signaling, it might be tenable that hsa_circ_0000199-miR-206/miR-613 axis controlled proliferation, migration, invasion, drug-resistance and autophagy of TNBC cells.

## CONCLUSIONS

**Figure 8:**
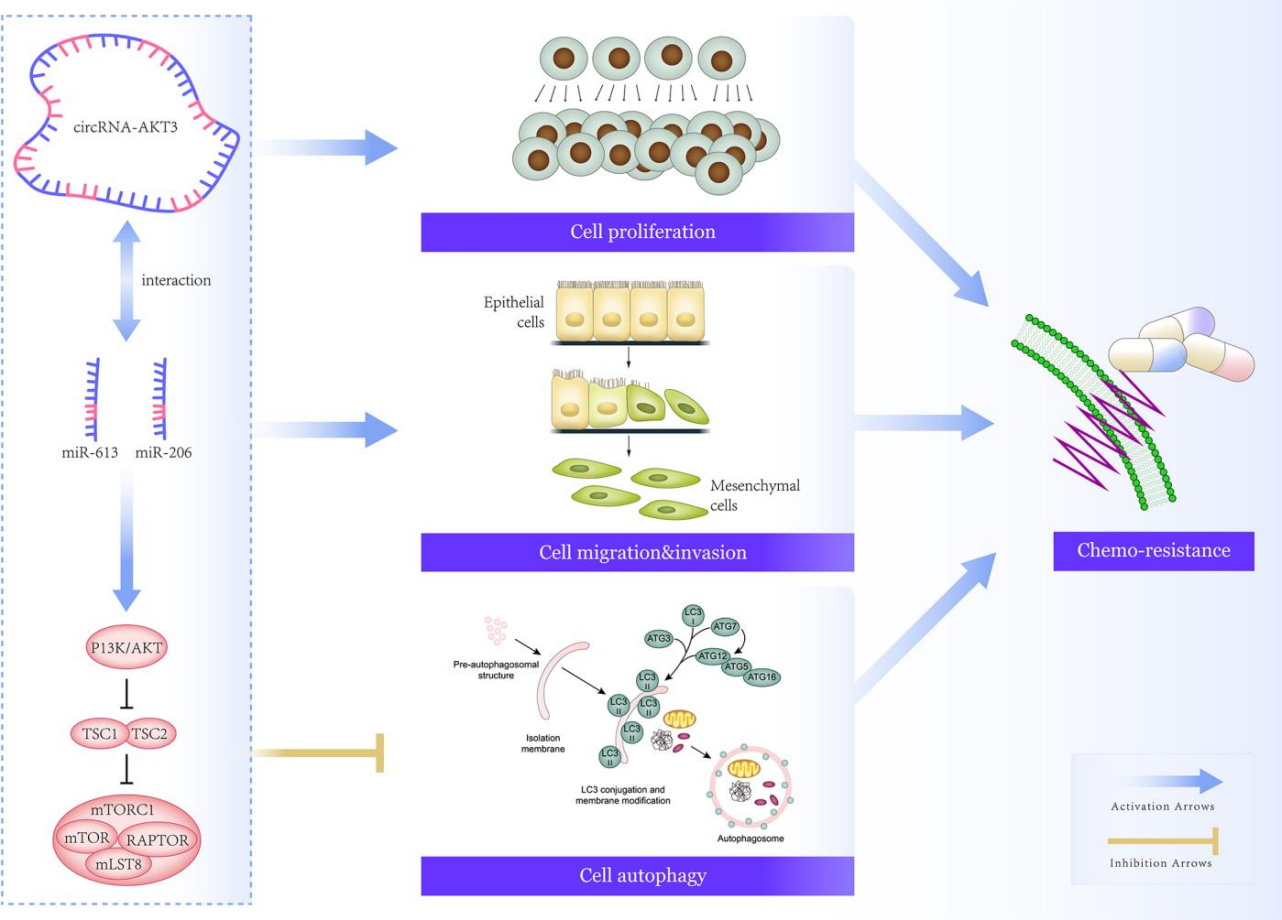
**Mechanism map that elaborated on involvement of hsa_circ_0000199-miR-613/miR-206 axis in regulating chemo-sensitivity of triple-negative breast cancer (TNBC) cells by modulation of PI3K/AKT/mTOR signaling.**

In conclusion, hsa_circ_0000199-miR-206/miR-613 axis pronouncedly disordered migration, invasion, chemo-resistance and autophagy of TNBC cells by motivating PI3K/Akt/mTOR signaling (Figure 8), providing molecular foundations for developing TNBC treatments. However, several pitfalls should be addressed in later researches. Firstly, it was uncertain whether hsa_circ_0000199 was applicable in distinguishing TNBC from other BC subtypes among populations of other ethnicities. Secondly, although hsa_circ_0000199 expression was heightened in tumor tissues of TNBC-bearing mice models as compared with paired normal tissues (Supplementary Figure 3), we failed to uncover the effect of over/under-expressed hsa_circ_0000199 on tumor formation in TNBC mice models owing to technical obstacles. Last but not the least, miRNA networks that aided hsa_circ_0000199 to function oncogenetically in TNBC should be expanded, so as to deepen understanding of TNBC pathogenesis. Above all, all these challenges should be coped with in future.

## MATERIALS AND METHODS

### Collection of BC samples

Two hundred and seven cases out of 210 primary BC patients (response rate: 98.57%) were recruited in Minhang Hospital affiliated to Fudan University, from December, 2012 to May, 2016. They were divided into TNBC group (n=83) and non-TNBC group (n=124) based on the immunohistochemical results, and they all underwent none of surgical puncture, immunity enhancement, chemotherapy and radiotherapy prior to surgery. The TNBC patients all exhibited negative expressions of ER, PR and Her-2, and the BC cases were graded according to TNM staging system revised by American Joint Committee on Cancer (AJCC)/Union International Center of Cancer (UICC) (6^th^ edition) [60]. The participants have signed informed consents, and this program was approved by Minhang Hospital affiliated to Fudan University and the ethics committee of Minhang Hospital affiliated to Fudan University. Additionally, BC tissues and adjacent normal tissues, after excision from patients during surgery, were split into pieces weighing around 0.1 g before storage at -80° C.

### Cell culture

Human mammary epithelial cell line (i.e. MCF-10A) was incubated in MEBM medium (Lonzo, Sweden), and human BC cell lines, including MDA-MB-231, MDA-MB-468, SK-BR-3 and MCF-7, were cultivated in RPMI 1640 medium (Hyclone, USA) which contained 10% fetal bovine serum (FBS) (Gibco, USA), 100 Ku/L penicillin (Solarbio, China) and 100 mg/L streptomycin (Solarbio, China). The cell lines were all purchased from American Type Culture Collection (ATCC, USA), and they were cultured under circumstances of 37° C, 5% CO_2_ and saturated humidity until 80%~90% confluence.

### Cell transfection

SiRNA against hsa_circ_0000199, si-negative control (NC), miR-206 inhibitor, miR-206 mimic, miR-613 inhibitor, miR-613 mimic and miR-NC were designed and synthesized by Geenseed Biotech corporation (Guangzhou, China). They were transfected into MDA-MB-231 and MDA-MB-468 cell lines for 48 h, strictly in line with the requirements of Lipofectamine^TM^ 2000 kit (Invitrogen, USA).

### Cell treatment by 3-methyladenine (3-MA)

TNBC cell lines after 48-h transfection were treated by 5 mmol /L 3-MA (Selleck, USA) for 24 h.

### Methyl thiazolyl tetrazolium (MTT) assay to assess chemosensitivity of TNBC cells

MDA-MB-231 and MDA-MB-468 cell lines of logarithmic growth were seeded into 96-well plates at the concentration of 1×10^4^ per well. After treatment by different concentrations of cisplatin (Shandong Qilu Pharmaceutical, China), adriamycin (Solarbio, China), paclitaxel (Bristol-Myers Squibb Company, USA) and gemcitabine (Eli Lily and Company, USA) for 48 h, the TNBC cell lines were incubated by MTT solution (Beyotime bIotechnology, China) for 4 h. Optical density (OD) of the cell lines was measured on the microplate reader (model: iMark, BioRad, USA) at the wavelength of 570 nm, and proliferation inhibition rate was calculated in line with the formula of. $\left(\frac{\text{1-OD test group}}{\text{OD control group}}\right)\times 100\%.$ The half maximal inhibitory concentration (IC50) values of each drug for each cell were calculated with online Quest Graph™ IC50 Calculator (https://www.aatbio.com/tools/ic50-calculator, AAT Bioquest, USA).

### Cell counting kit-8 (CCK8) assay

Abiding by procedures detailed in the CCK8 kit (Sino-American Biotechnology, China), MDA-MB-231 and MDA-MB-468 cell lines were blended with 10 μl enhanced CCK8 reagent. After incubation at 37° C for 1 h, optical density (OD) of TNBC cells was evaluated on microplate reader (model: iMark, BioRad, USA) at the wavelength of 450 nm.

### Transwell assays

#### Cell migration

The upper transwell chamber (Corning, USA) was inoculated by 1×10^5^ TNBC cells, and 700 μl 10% FBS-containing DMEM (Biological Industries, USA) was supplemented into the lower transwell chamber. After cultivation in 5% CO_2_ for 24 h, MDA-MB-231 and MDA-MB-468 cell lines were successively fixated by 10% methanol for 20 min and stained by 0.5% crystal violet for 30 min. TNBC cells left on the membrane were eliminated with a cotton rub, and pictures of 5 views were taken to count average cell number with inverted microscope (Nikon, Japan).

#### Cell invasion

After dilution by serum-free and high-glucose DMEM (Biological Industries, USA) at the ratio of 1:8, 100 μl Matrigel (Corning, USA) was paved onto the center of upper transwell chamber (Corning, USA). Then 1×10^5^ TNBC cells were supplemented onto the coagulated Matrigel, and 700 μl 10% FBS-inclusive high-glucose DMEM was poured into the lower chamber (Corning, USA). Twenty-four hours later, MDA-MB-231 and MDA-MB-468 cell lines were managed by 10% methanol for 20 min, followed by dyeing with 0.5% crystal violet for 30 min. Eventually, TNBC cells that hardly penetrated the upper chamber were wiped off, and 5 fields were randomly selected to count cell number under inverted microscope (Nikon, Japan).

#### Dual-luciferase reporter gene assay

Hsa_circ_000019 fragments that included binding sites with miR-613 or miR-206 were, respectively, mutated, and the products were integrated into pGL3 vector (Promega, USA) to construct pGL3-hsa_circ_0000199-Mut-1 and pGL3-hsa_circ_0000199-Mut-2. Simultaneously, pGL3-hsa_circ_0000199-Wt-1 and pGL3-hsa_circ_0000199-Wt-2 were constructed by amplifying hsa_circ_000019 fragments which contained binding sites with miR-613 and miR-206. Afterwards, MDA-MB-231 and MDA-MB-468 cell lines were, respectively, transfected by: 1) miR-613 mimic+hsa_circ_0000199-Wt-1, 2) miR-NC+hsa_circ_0000199-Wt-1, 3) miR-613 mimic+hsa_circ_0000199-Mut-1, 4) miR-NC+-hsa_circ_0000199-Mut-1, 5) miR-206 mimic+hsa_circ_0000199-Wt-2, 6) miR-NC+hsa_circ_0000199-Wt-2, 7) miR-206 mimic+hsa_circ_0000199-Mut-2, and 8) miR-NC+ hsa_circ_0000199-Mut-2, as per specifications of Lipofectamine^TM^ 2000 kit (Invitrogen, USA). Twenty-four hours later, MDA-MB-231 and MDA-MB-468 cells of each group were lysed, and the mixture was centrifuged at 3000×g for 5 min. After removal of supernatants, luciferase activity of each sample, designated as the ratio of Firefly luciferase activity and Renilla luciferase activity, was examined with Dual-luciferase assay kit (Promega, USA).

#### Monodansylcadaverine (MDC) staining to determine autophagic condition of TNBC cells

After digestion by pancreatin to a density of 3×10^4^/ml, MDA-MB-231 and MDA-MB-468 cell lines at the logarithmic growth phase were inoculated into 12-well plates until 70% confluence. Each cell sample was evenly mixed with 10 μl MDC solution (Sigma, USA), which was then left in the darkness for 40 min. After centrifugation at 1000 g for 5 min, the TNBC cells were re-suspended in 100 μl PBS, and they were photographed under fluorescent microscope (Olympus, Japan).

#### Real-time quantitative polymerase chain reaction (PCR)

Total RNAs were extracted from BC tissues and cell lines by addition of TRIzol reagent (Invitrogen, USA). Integrity of the RNAs was confirmed through agarose gel electrophoresis (AGE), and their concentration and purity were determined with spectrophotometer (model: SmartSpec Plus, Bio-Rad, USA). Subsequently, the RNAs were reversely transcribed into cDNAs (TransGen Biotech, China), and cDNAs were amplified (Applied Biosystems, USA) on the real-time PCR instrument (model: 9300, Bio-Rad, USA) following procedures of: 1) pre-denaturation at 95° C for 3 min, and 2) 40 cycles of denaturation at 95° C for 5 s, annealing at 60° C for 30 s and extension at 72° C for 30 s. Primers of circRNAs and miRNAs were arranged in Supplementary Tables 1, 2, and their relative expression was calculated through 2^-ΔΔCt^ approach [61]. Expressions of circRNAs were normalized to that of GAPDH, and U6 was set as the internal reference of miRNAs.

#### Western blotting

MDA-MB-231 and MDA-MB-468 cell lines of logarithmic growth phase, after digestion by 0.25% trypsin to a density of 5×10^5^/ml, were inoculated into 96-well plates. After dissociation by 200 μl RIPA at 4° C for 30 min, TNBC cells were centrifuged at 12000×g for 30 min, and supernatants were collected to quantify proteins through Bradford method (Bio-Rad, USA). Subsequently, 20 μg of each protein sample was separated to carry out sodium dodecyl sulfate-polyacrylamide gel electrophoresis (SDS-PAGE) (Bio-Rad, USA), and the products were shifted onto polyvinylidene fluoride (PVDF) membrane (EMD Millipore, USA). In the wake of blockage within 5% skimmed milk (v/v) for 2 h, primary antibodies (rabbit anti-human, Abcam, USA) against beclin 1 (1:2000, ab207612), LC3-II (1:200, ab222776), p62 (1:2000, ab101266), Atg5 (1:5000, ab199560), PI3K (1:1000, ab191606), p-PI3K (rabbit anti-human, 1:1000, ab182651), Akt (rabbit anti-human, 1:10000, ab179463), p-Akt (rabbit anti-human, 1:500, ab38449), mTOR (rabbit anti-human, 1:10000, ab134903) and p-mTOR (rabbit anti-human, 1:1000, ab109268) were formulated to incubate protein samples at 4° C for overnight. After rinsage with TBST for 3 times (5 min each time), samples were incubated by IgG H&L (HRP) secondary antibody (goat-anti-rabbit, 1:10000, ab97080, Abcam, USA) for 1 h, and they were analyzed by virtue of electro-chemiluminescence (ECL) imaging system (Thermo, USA).

#### Statistical analyses

All data were analyzed with SPSS 17.0 software (IBM Corporation, USA). Differences among categorical variables (n) were discerned using chi-square test, and continuous variables [mean ± standard deviation (SD)] were compared through student’s t test or single-factor analysis of variance (ANOVA). Kaplan-Meier survival curves were plotted, and log-rank test was applied to identify statistical difference between groups. Cox-proportional hazard model was also established to screen out variables that were predictive of TNBC patients’ survival. It was statistically significant when *P* value was less than 0.05.

#### Ethics approval and consent to participate

All these operations and experimental process have been approved by the ethics committee and the experimental animal ethics committee of Minhang Hospital affiliated to Fudan University.

#### Availability of data and materials

All data generated or analyzed during this study are included in this article.

## Supplementary Material

Supplementary Figures

Supplementary Tables

## References

[1] Li N, Deng Y, Zhou L, Tian T, Yang S, Wu Y, Zheng Y, Zhai Z, Hao Q, Song D, Zhang D, Kang H, Dai Z. Global burden of breast cancer and attributable risk factors in 195 countries and territories, from 1990 to 2017: results from the Global Burden of Disease Study 2017. J Hematol Oncol. 2019; 12:140. 10.1186/s13045-019-0828-0 31864424 PMC6925497

[2] Greenup R, Buchanan A, Lorizio W, Rhoads K, Chan S, Leedom T, King R, McLennan J, Crawford B, Kelly Marcom P, Shelley Hwang E. Prevalence of BRCA mutations among women with triple-negative breast cancer (TNBC) in a genetic counseling cohort. Ann Surg Oncol. 2013; 20:3254–58. 10.1245/s10434-013-3205-1 23975317

[3] Asano Y, Kashiwagi S, Onoda N, Noda S, Kawajiri H, Takashima T, Ohsawa M, Kitagawa S, Hirakawa K. Predictive value of neutrophil/lymphocyte ratio for efficacy of preoperative chemotherapy in triple-negative breast cancer. Ann Surg Oncol. 2016; 23:1104–10. 10.1245/s10434-015-4934-0 26511266 PMC4773470

[4] Bianchini G, Balko JM, Mayer IA, Sanders ME, Gianni L. Triple-negative breast cancer: challenges and opportunities of a heterogeneous disease. Nat Rev Clin Oncol. 2016; 13:674–90. 10.1038/nrclinonc.2016.66 27184417 PMC5461122

[5] Zhang HD, Jiang LH, Sun DW, Hou JC, Ji ZL. CircRNA: a novel type of biomarker for cancer. Breast Cancer. 2018; 25:1–7. 10.1007/s12282-017-0793-9 28721656

[6] Qu Y, Dou P, Hu M, Xu J, Xia W, Sun H. circRNA-CER mediates Malignant progression of breast cancer through targeting the miR-136/MMP13 axis. Mol Med Rep. 2019; 19:3314–20. 10.3892/mmr.2019.9965 30816475

[7] Li R, Jiang J, Shi H, Qian H, Zhang X, Xu W. CircRNA: a rising star in gastric cancer. Cell Mol Life Sci. 2020; 77:1661–80. 10.1007/s00018-019-03345-5 31659415 PMC11104848

[8] Cao Q, Shi Y, Wang X, Yang J, Mi Y, Zhai G, Zhang M. Circular METRN RNA hsa_circ_0037251 promotes glioma progression by sponging miR-1229-3p and regulating mTOR expression. Sci Rep. 2019; 9:19791. 10.1038/s41598-019-56417-8 31875034 PMC6930248

[9] Min L, Wang H, Zeng Y. CircRNA_104916 regulates migration, apoptosis and epithelial-mesenchymal transition in colon cancer cells. Front Biosci (Landmark Ed). 2019; 24:819–32. 30844715 10.2741/4753

[10] Tang H, Huang X, Wang J, Yang L, Kong Y, Gao G, Zhang L, Chen ZS, Xie X. circKIF4A acts as a prognostic factor and mediator to regulate the progression of triple-negative breast cancer. Mol Cancer. 2019; 18:23. 10.1186/s12943-019-0946-x 30744636 PMC6369546

[11] Yang R, Xing L, Zheng X, Sun Y, Wang X, Chen J. The circRNA circAGFG1 acts as a sponge of miR-195-5p to promote triple-negative breast cancer progression through regulating CCNE1 expression. Mol Cancer. 2019; 18:4. 10.1186/s12943-018-0933-7 30621700 PMC6325825

[12] He R, Liu P, Xie X, Zhou Y, Liao Q, Xiong W, Li X, Li G, Zeng Z, Tang H. circGFRA1 and GFRA1 act as ceRNAs in triple negative breast cancer by regulating miR-34a. J Exp Clin Cancer Res. 2017; 36:145. 10.1186/s13046-017-0614-1 29037220 PMC5644184

[13] Gao D, Zhang X, Liu B, Meng D, Fang K, Guo Z, Li L. Screening circular RNA related to chemotherapeutic resistance in breast cancer. Epigenomics. 2017; 9:1175–88. 10.2217/epi-2017-0055 28803498

[14] Chen L, Zhou H, Guan Z. CircRNA_000543 knockdown sensitizes nasopharyngeal carcinoma to irradiation by targeting miR-9/platelet-derived growth factor receptor B axis. Biochem Biophys Res Commun. 2019; 512:786–92. 10.1016/j.bbrc.2019.03.126 30928094

[15] Shuai M, Hong J, Huang D, Zhang X, Tian Y. Upregulation of circRNA_0000285 serves as a prognostic biomarker for nasopharyngeal carcinoma and is involved in radiosensitivity. Oncol Lett. 2018; 16:6495–501. 10.3892/ol.2018.9471 30405788 PMC6202549

[16] Xiong H, Yan T, Zhang W, Shi F, Jiang X, Wang X, Li S, Chen Y, Chen C, Zhu Y. miR-613 inhibits cell migration and invasion by downregulating Daam1 in triple-negative breast cancer. Cell Signal. 2018; 44:33–42. 10.1016/j.cellsig.2018.01.013 29339084

[17] Xia T, Liao Q, Jiang X, Shao Y, Xiao B, Xi Y, Guo J. Long noncoding RNA associated-competing endogenous RNAs in gastric cancer. Sci Rep. 2014; 4:6088. 10.1038/srep06088 25124853 PMC4133709

[18] Madhunapantula SV, Robertson GP. Targeting protein kinase-b3 (Akt3) signaling in melanoma. Expert Opin Ther Targets. 2017; 21:273–90. 10.1080/14728222.2017.1279147 28064546

[19] Banerji S, Cibulskis K, Rangel-Escareno C, Brown KK, Carter SL, Frederick AM, Lawrence MS, Sivachenko AY, Sougnez C, Zou L, Cortes ML, Fernandez-Lopez JC, Peng S, et al. Sequence analysis of mutations and translocations across breast cancer subtypes. Nature. 2012; 486:405–09. 10.1038/nature11154 22722202 PMC4148686

[20] Zhuang J, Ye Y, Wang G, Ni J, He S, Hu C, Xia W, Lv Z. MicroRNA-497 inhibits cellular proliferation, migration and invasion of papillary thyroid cancer by directly targeting AKT3. Mol Med Rep. 2017; 16:5815–22. 10.3892/mmr.2017.7345 28849051 PMC5865779

[21] Grabinski N, Möllmann K, Milde-Langosch K, Müller V, Schumacher U, Brandt B, Pantel K, Jücker M. AKT3 regulates ErbB2, ErbB3 and estrogen receptor α expression and contributes to endocrine therapy resistance of ErbB2(+) breast tumor cells from Balb-neuT mice. Cell Signal. 2014; 26:1021–29. 10.1016/j.cellsig.2014.01.018 24463007

[22] Hansen TB, Jensen TI, Clausen BH, Bramsen JB, Finsen B, Damgaard CK, Kjems J. Natural RNA circles function as efficient microRNA sponges. Nature. 2013; 495:384–88. 10.1038/nature11993 23446346

[23] Liu T, Ye P, Ye Y, Lu S, Han B. Circular RNA hsa_circRNA_002178 silencing retards breast cancer progression via microRNA-328-3p-mediated inhibition of COL1A1. J Cell Mol Med. 2020; 24:2189–201. 10.1111/jcmm.14875 31957232 PMC7011152

[24] Li JH, Liu S, Zhou H, Qu LH, Yang JH. starBase v2.0: decoding miRNA-ceRNA, miRNA-ncRNA and protein-RNA interaction networks from large-scale CLIP-Seq data. Nucleic Acids Res. 2014; 42:D92–97. 10.1093/nar/gkt1248 24297251 PMC3964941

[25] Dudekula DB, Panda AC, Grammatikakis I, De S, Abdelmohsen K, Gorospe M. CircInteractome: a web tool for exploring circular RNAs and their interacting proteins and microRNAs. RNA Biol. 2016; 13:34–42. 10.1080/15476286.2015.1128065 26669964 PMC4829301

[26] Vlachos IS, Zagganas K, Paraskevopoulou MD, Georgakilas G, Karagkouni D, Vergoulis T, Dalamagas T, Hatzigeorgiou AG. DIANA-miRPath v3.0: deciphering microRNA function with experimental support. Nucleic Acids Res. 2015; 43:W460–66. 10.1093/nar/gkv403 25977294 PMC4489228

[27] Wang J, Tsouko E, Jonsson P, Bergh J, Hartman J, Aydogdu E, Williams C. miR-206 inhibits cell migration through direct targeting of the actin-binding protein coronin 1C in triple-negative breast cancer. Mol Oncol. 2014; 8:1690–702. 10.1016/j.molonc.2014.07.006 25074552 PMC5528580

[28] Saiki S, Sasazawa Y, Imamichi Y, Kawajiri S, Fujimaki T, Tanida I, Kobayashi H, Sato F, Sato S, Ishikawa K, Imoto M, Hattori N. Caffeine induces apoptosis by enhancement of autophagy via PI3K/Akt/mTOR/p70S6K inhibition. Autophagy. 2011; 7:176–87. 10.4161/auto.7.2.14074 21081844 PMC3039768

[29] Zhang Q, Yang W, Man N, Zheng F, Shen Y, Sun K, Li Y, Wen LP. Autophagy-mediated chemosensitization in cancer cells by fullerene C60 nanocrystal. Autophagy. 2009; 5:1107–17. 10.4161/auto.5.8.9842 19786831

[30] Meschini S, Condello M, Marra M, Formisano G, Federici E, Arancia G. Autophagy-mediated chemosensitizing effect of the plant alkaloid voacamine on multidrug resistant cells. Toxicol In Vitro. 2007; 21:197–203. 10.1016/j.tiv.2006.09.007 17070665

[31] Weh KM, Howell AB, Kresty LA. Expression, modulation, and clinical correlates of the autophagy protein Beclin-1 in esophageal adenocarcinoma. Mol Carcinog. 2016; 55:1876–85. 10.1002/mc.22432 27696537 PMC5987534

[32] Li DD, Wang LL, Deng R, Tang J, Shen Y, Guo JF, Wang Y, Xia LP, Feng GK, Liu QQ, Huang WL, Zeng YX, Zhu XF. The pivotal role of c-Jun NH2-terminal kinase-mediated Beclin 1 expression during anticancer agents-induced autophagy in cancer cells. Oncogene. 2009; 28:886–98. 10.1038/onc.2008.441 19060920

[33] Pankiv S, Clausen TH, Lamark T, Brech A, Bruun JA, Outzen H, Øvervatn A, Bjørkøy G, Johansen T. p62/SQSTM1 binds directly to Atg8/LC3 to facilitate degradation of ubiquitinated protein aggregates by autophagy. J Biol Chem. 2007; 282:24131–45. 10.1074/jbc.M702824200 17580304

[34] Peng J, Zhang R, Cui Y, Liu H, Zhao X, Huang L, Hu M, Yuan X, Ma B, Ma X, Takashi U, Masaaki K, Liang X, Yu L. Atg5 regulates late endosome and lysosome biogenesis. Sci China Life Sci. 2014; 57:59–68. 10.1007/s11427-013-4588-8 24369351

[35] Siegel RL, Miller KD, Jemal A. Cancer statistics, 2019. CA Cancer J Clin. 2019; 69:7–34. 10.3322/caac.21551 30620402

[36] Kumar P, Aggarwal R. An overview of triple-negative breast cancer. Arch Gynecol Obstet. 2016; 293:247–69. 10.1007/s00404-015-3859-y 26341644

[37] Geng Y, Jiang J, Wu C. Function and clinical significance of circRNAs in solid tumors. J Hematol Oncol. 2018; 11:98. 10.1186/s13045-018-0643-z 30064463 PMC6069963

[38] Zhong Z, Huang M, Lv M, He Y, Duan C, Zhang L, Chen J. Circular RNA MYLK as a competing endogenous RNA promotes bladder cancer progression through modulating VEGFA/VEGFR2 signaling pathway. Cancer Lett. 2017; 403:305–17. 10.1016/j.canlet.2017.06.027 28687357

[39] Qin M, Liu G, Huo X, Tao X, Sun X, Ge Z, Yang J, Fan J, Liu L, Qin W. Hsa_circ_0001649: a circular RNA and potential novel biomarker for hepatocellular carcinoma. Cancer Biomark. 2016; 16:161–69. 10.3233/CBM-150552 26600397 PMC13016540

[40] Xuan L, Qu L, Zhou H, Wang P, Yu H, Wu T, Wang X, Li Q, Tian L, Liu M, Sun Y. Circular RNA: a novel biomarker for progressive laryngeal cancer. Am J Transl Res. 2016; 8:932–39. 27158380 PMC4846937

[41] Sand M, Bechara FG, Sand D, Gambichler T, Hahn SA, Bromba M, Stockfleth E, Hessam S. Circular RNA expression in basal cell carcinoma. Epigenomics. 2016; 8:619–32. 10.2217/epi-2015-0019 27097056

[42] Lü L, Sun J, Shi P, Kong W, Xu K, He B, Zhang S, Wang J. Identification of circular RNAs as a promising new class of diagnostic biomarkers for human breast cancer. Oncotarget. 2017; 8:44096–107. 10.18632/oncotarget.17307 28484086 PMC5546465

[43] Liang G, Liu Z, Tan L, Su AN, Jiang WG, Gong C. HIF1α-associated circDENND4C promotes proliferation of breast cancer cells in hypoxic environment. Anticancer Res. 2017; 37:4337–43. 10.21873/anticanres.11827 28739726

[44] Chen B, Wei W, Huang X, Xie X, Kong Y, Dai D, Yang L, Wang J, Tang H, Xie X. circEPSTI1 as a prognostic marker and mediator of triple-negative breast cancer progression. Theranostics. 2018; 8:4003–15. 10.7150/thno.24106 30083277 PMC6071524

[45] Chen J, Xu J, Li Y, Zhang J, Chen H, Lu J, Wang Z, Zhao X, Xu K, Li Y, Li X, Zhang Y. Competing endogenous RNA network analysis identifies critical genes among the different breast cancer subtypes. Oncotarget. 2017; 8:10171–84. 10.18632/oncotarget.14361 28052038 PMC5354650

[46] Uhr K, Sieuwerts AM, de Weerd V, Smid M, Hammerl D, Foekens JA, Martens JW. Association of microRNA-7 and its binding partner CDR1-AS with the prognosis and prediction of 1^st^-line tamoxifen therapy in breast cancer. Sci Rep. 2018; 8:9657. 10.1038/s41598-018-27987-w 29941867 PMC6018428

[47] Yin K, Yin W, Wang Y, Zhou L, Liu Y, Yang G, Wang J, Lu J. MiR-206 suppresses epithelial mesenchymal transition by targeting TGF-β signaling in estrogen receptor positive breast cancer cells. Oncotarget. 2016; 7:24537–48. 10.18632/oncotarget.8233 27014911 PMC5029720

[48] Xiang Y, Liao XH, Yao A, Qin H, Fan LJ, Li JP, Hu P, Li H, Guo W, Li JY, Gu CJ, Bao LY, Zhang TC. MRTF-A-miR-206-WDR1 form feedback loop to regulate breast cancer cell migration. Exp Cell Res. 2017; 359:394–404. 10.1016/j.yexcr.2017.08.023 28822708

[49] Ding D, Hou R, Gao Y, Feng Y. miR-613 inhibits gastric cancer progression through repressing brain derived neurotrophic factor. Exp Ther Med. 2018; 15:1735–41. 10.3892/etm.2017.5546 29434759 PMC5774479

[50] Yu H, Duan P, Zhu H, Rao D. miR-613 inhibits bladder cancer proliferation and migration through targeting SphK1. Am J Transl Res. 2017; 9:1213–21. 28386347 PMC5376012

[51] Zhu Y, Tang L, Zhao S, Sun B, Cheng L, Tang Y, Luo Z, Lin Z, Zhu J, Zhu W, Zhao R, Lu B, Long H. CXCR4-mediated osteosarcoma growth and pulmonary metastasis is suppressed by MicroRNA-613. Cancer Sci. 2018; 109:2412–22. 10.1111/cas.13653 29845707 PMC6113448

[52] Qiu W, Yang Z, Fan Y, Zheng Q. MicroRNA-613 inhibits cell growth, migration and invasion of papillary thyroid carcinoma by regulating SphK2. Oncotarget. 2016; 7:39907–15. 10.18632/oncotarget.9530 27223438 PMC5129980

[53] Yang X, Zhang L, Song X, He W, Zhang D, Lu Q, Wu J, Wu C, Jiang J. MicroRNA-613 promotes colon cancer cell proliferation, invasion and migration by targeting ATOH1. Biochem Biophys Res Commun. 2018; 504:827–33. 10.1016/j.bbrc.2018.09.054 30219232

[54] Massihnia D, Galvano A, Fanale D, Perez A, Castiglia M, Incorvaia L, Listì A, Rizzo S, Cicero G, Bazan V, Castorina S, Russo A. Triple negative breast cancer: shedding light onto the role of pi3k/Akt/mtor pathway. Oncotarget. 2016; 7:60712–22. 10.18632/oncotarget.10858 27474173 PMC5312414

[55] Walsh S, Flanagan L, Quinn C, Evoy D, McDermott EW, Pierce A, Duffy MJ. mTOR in breast cancer: differential expression in triple-negative and non-triple-negative tumors. Breast. 2012; 21:178–82. 10.1016/j.breast.2011.09.008 21963359

[56] Dai W, Yang F, Ma L, Fan Y, He B, He Q, Wang X, Zhang H, Zhang Q. Combined mTOR inhibitor rapamycin and doxorubicin-loaded cyclic octapeptide modified liposomes for targeting integrin α3 in triple-negative breast cancer. Biomaterials. 2014; 35:5347–58. 10.1016/j.biomaterials.2014.03.036 24726747

[57] Tomao F, Papa A, Zaccarelli E, Rossi L, Caruso D, Minozzi M, Vici P, Frati L, Tomao S. Triple-negative breast cancer: new perspectives for targeted therapies. Onco Targets Ther. 2015; 8:177–93. 10.2147/OTT.S67673 25653541 PMC4303459

[58] Wirawan E, Vanden Berghe T, Lippens S, Agostinis P, Vandenabeele P. Autophagy: for better or for worse. Cell Res. 2012; 22:43–61. 10.1038/cr.2011.152 21912435 PMC3351915

[59] Han J, Han B, Wu X, Hao J, Dong X, Shen Q, Pang H. Knockdown of lncRNA H19 restores chemo-sensitivity in paclitaxel-resistant triple-negative breast cancer through triggering apoptosis and regulating Akt signaling pathway. Toxicol Appl Pharmacol. 2018; 359:55–61. 10.1016/j.taap.2018.09.018 30244121

[60] Singletary SE, Allred C, Ashley P, Bassett LW, Berry D, Bland KI, Borgen PI, Clark GM, Edge SB, Hayes DF, Hughes LL, Hutter RV, Morrow M, et al. Staging system for breast cancer: revisions for the 6^th^ edition of the AJCC cancer staging manual. Surg Clin North Am. 2003; 83:803–19. 10.1016/S0039-6109(03)00034-3 12875597

[61] Reid G, Kirschner MB, van Zandwijk N. Circulating microRNAs: association with disease and potential use as biomarkers. Crit Rev Oncol Hematol. 2011; 80:193–208. 10.1016/j.critrevonc.2010.11.004 21145252

