# Supplementary material for: Hsa_circ_0000199 facilitates chemo-tolerance of triple-negative breast cancer by interfering with miR-206/613-led PI3K/Akt/mTOR signaling
Source: Aging (Albany NY). 2021 Jan 20;13(3):4522–51. doi: 10.18632/aging.202415 (PMC7906206; doi:10.18632/aging.202415)
Supplement: Supplementary Figures [file aging-13-202415-s001.pdf]

SUPPLEMENTARY FIGURES

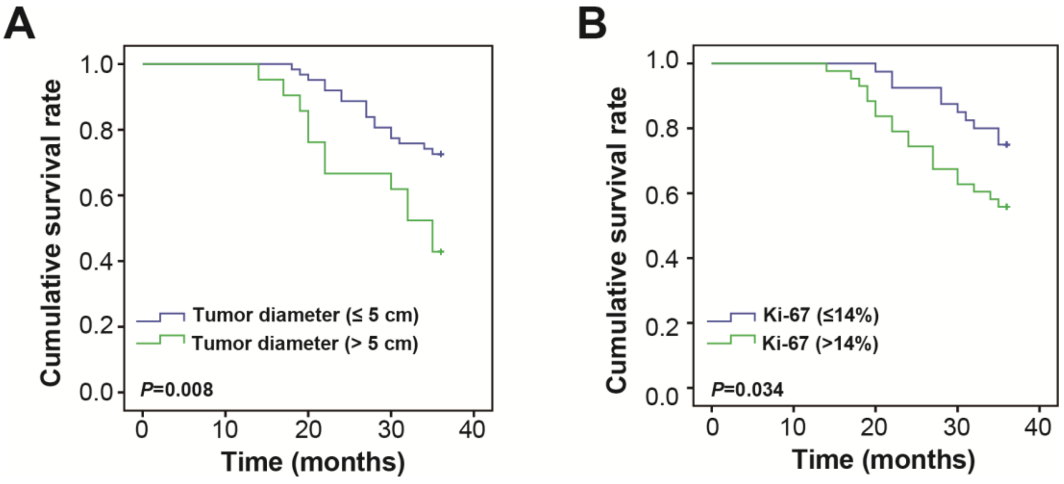

Supplementary Figure 1. Large tumor size (**A**) and high Ki-67 proportion (**B**) were associated with poor survival of triple-negative breast cancer (TNBC) patients.

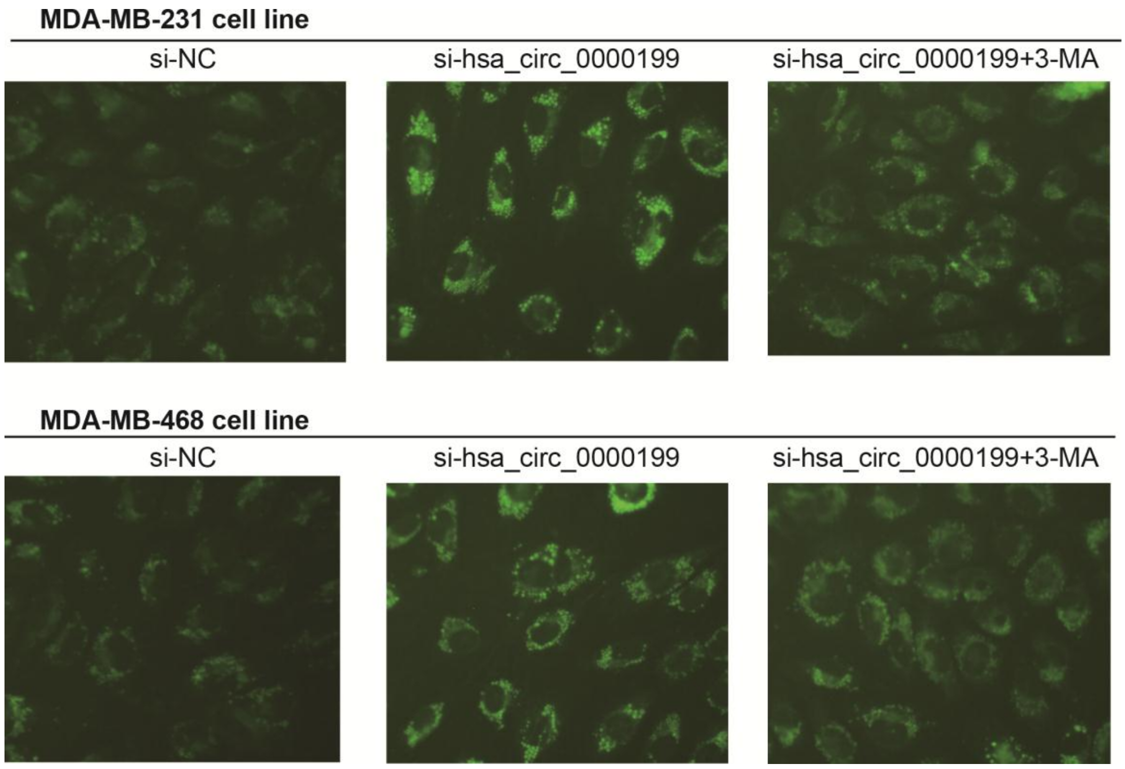

Supplementary Figure 2. Comparison of Monodansylcadaverine (MDC) fluorescence intensity among MDA-MB-231 and MDA-MB-468 cell lines under treatments of si-NC, si-hsa_circ_0000199 and si-hsa_circ_0000199+3-MA. 3-MA: 3-methyladenine.

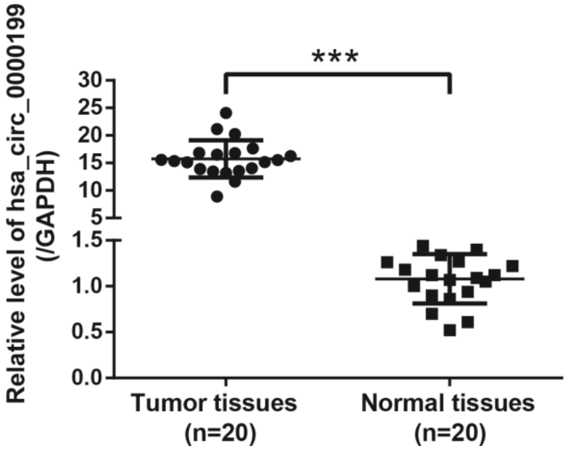

Supplementary Figure 3. Hsa_circ_0000199 expression was compared between tumor tissues and normal tissues in triple-negative breast cancer (TNBC)-bearing mice models. ***: *P*<0.001. #Establishment of TNBC-bearing mice models: Single-cell suspension of MDA-MB-231 cell line (concentration: 1.5×10^{7}/ml) was injected into the subcutaneous fat pad in the chest of SPF-degree female BALB/Cnu/nu mice (n=20), which were provided by Laboratory Animal department of Minhang Hospital affiliated to Fudan University. The mice were aged 5-6 weeks old, and their weight ranged from 18 g to 20 g. On the 2nd day after inoculation, nodes were observable at their injection site. Ten days later, tumors were formed in all mice models, whose tumor tissues and normal tissues were then excised to determine hsa_circ_0000199 expression utilizing PCR. All the procedures were finished in accordance with requirements of Care and Use of Experimental Animals of the US National Institutes of Health (Bethesda, USA), and approvals were obtained from Animal Care Committee of Minhang Hospital affiliated to Fudan University.
