# Supplementary material for: Hsa_circ_0000199 facilitates chemo-tolerance of triple-negative breast cancer by interfering with miR-206/613-led PI3K/Akt/mTOR signaling
Source: Aging (Albany NY). 2021 Jan 20;13(3):4522–51. doi: 10.18632/aging.202415 (PMC7906206; doi:10.18632/aging.202415)
Supplement: Supplementary Tables [file aging-13-202415-s002.pdf]

SUPPLEMENTARY TABLES

Supplementary Table 1. Primers for circRNAs and their internal reference.

Genes	Primers	
	Forward	Reverse
hsa_circ_0017242	5'-TTGGTGGAGGACCAGATGAT-3'	5'-CCCCCAACTTGGAGAAATGGTA-3'
hsa_circ_0017251	5'-TCCTTCCAGACAAAAGACCGTT-3'	5'-TCCACATCTTGAGGTTTCTCTT-3'
hsa_circ_0006696	5'-TCCTTCCAGACAAAAGACCGT-3'	5'-TCCACTTGCCTTCTCTCGAAC-3'
hsa_circ_0017252	5'-CCTTCCAGACAAAAGACCGT-3'	5'-CCCCCAACTTGGAGAAATGGT-3'
hsa_circ_0017243	5'-GCAGCCACCATGAAGACATTC-3'	5'-AGGTAAATCCACATCTTGAGGTT-3'
hsa_circ_0004649	5'-TGAAGACAGATGGCTCATTCAT-3'	5'-GGTTTGGATTCTCTGCTGCT-3'
hsa_circ_0017254	5'-GGATGCCTCTACAACCCATC-3'	5'-CCCCAACTTGGAGAAATGGT-3'
hsa_circ_0017246	5'-ACCGCACACGTTTCTATGGT-3'	5'-ATGTGTTTGGCTTTGGTCGT-3'
hsa_circ_0017250	5'-CATCCCTTTTTAACATCCTTGAA-3'	5'-GTGTTTGGCTTTGGTCGTTC-3'
hsa_circ_0000199	5'-CAAATAAACGCCTTGGTGGA-3'	5'-ATAGAAACGTGTGCGGTCCT-3'
hsa_circ_0017247	5'-AGGACCGCACACGTTTCTAT-3'	5'-GGAAGTATCTTGGCCTCCAG-3'
hsa_circ_0017244	5'-TTTGCAAAGAAGGGATCACA-3'	5'-AGGGTTTGGATTCTCTGCTG-3'
hsa_circ_0017253	5'-TGGTTCGAGAGAAGGCAAGT-3'	5'-GGTTTGGATTCTCTGCTGCT-3'
hsa_circ_0002240	5'-CCTCCTTTTAAACCTCAAGTAACATC-3'	5'-ATAGAAACGTGTGCGGTCCT-3'
hsa_circ_0017245	5'-ACCGCACACGTTTCTATGGT-3'	5'-TCGCCCCCATTAACATATTC-3'
hsa_circ_0017248	5'-AGGACCGCACACGTTTCTAT-3'	5'-AGGGTTTGGATTCTCTGCTG-3'
GAPDH	5’-ACAACTTTGGTATCGTGGAAGG-3’	5’-GCCATCACGCCACAGTTTC-3’

Supplementary Table 2. Primers for miRNAs and their internal reference.

Genes	Primers	
	Forward	Reverse
miR-613	5'-GGCGAAAGGAATGTTCCTTCT-3'	5'-CAGTGCGTGTCGTGGAGT-3'
miR-1-3p	5'-GCGGCGGTGGAATGTAAAGAAG-3'	5'-ATCCAGTGCAGGGTCCGAGG-3'
miR-206	5'-CGTCAGAAGGAATGATGCACAG-3'	5'-ACCTGCGTAGGTAGTTTCATGT-3'
miR-147b	5'-GCGGCGGGTGTGCGGAAATGCTTC-3'	5'-ATCCAGTGCAGGGTCCGAGG-3'
miR-450b-5p	5'-GCGGCGGTTTTGCAATATGTTC-3'	5'-ATCCAGTGCAGGGTCCGAGG-3'
miR-942-5p	5'-GCGGCGGTCTTCTCTGTTTTGG-3'	5'-ATCCAGTGCAGGGTCCGAGG-3'
miR-20a-5p	5'-GCGGCGGTAAAGTGCTTATAGTG-3'	5'-ATCCAGTGCAGGGTCCGAGG-3'
miR-106b-5p	5'-GCGGCGGTAAAGTGCTGACAGTG-3'	5'-ATCCAGTGCAGGGTCCGAGG-3'
miR-20b-5p	5'-GCGGCGGCAAAGTGCTCATAGTGC-3'	5'-ATCCAGTGCAGGGTCCGAGG-3'
miR-526b-3p	5'-GCGGCGGGAAAGTGCTTCCTTT-3'	5'-ATCCAGTGCAGGGTCCGAGG-3'
miR-519d-3p	5'-GCGGCGGCAAAGTGCCTCCC-3'	5'-ATCCAGTGCAGGGTCCGAGG-3'
miR-93-5p	5'-GCGGCGGCAAAGTGCTGTTCGTG-3'	5'-ATCCAGTGCAGGGTCCGAGG-3'
miR-17-5p	5'-GCGGCGGCAAAGTGCTTACAGTG-3'	5'-ATCCAGTGCAGGGTCCGAGG-3'
miR-325	5'-GCGGCGGCCTAGTAGGTGTCCAG-3'	5'-ATCCAGTGCAGGGTCCGAGG-3'
miR-382-3p	5'-GCGGCGGAATCATTCACGGACAAC-3'	5'-ATCCAGTGCAGGGTCCGAGG-3'
miR-337-3p	5'-GCGGCGGCTCCTATATGATGCC-3'	5'-ATCCAGTGCAGGGTCCGAGG-3'
miR-656-3p	5'-GCGGCGGAATATTATACAGTCAAC-3'	5'-ATCCAGTGCAGGGTCCGAGG-3'
miR-3611	5'-GCGGCGGTTGTGAAGAAAGAAA-3'	5'-ATCCAGTGCAGGGTCCGAGG-3'
miR-516b-5p	5'-GCGGCGGATCTGGAGGTAAGAAG-3'	5'-ATCCAGTGCAGGGTCCGAGG-3'
miR-3129-5p	5'-GCGGCGGGCAGTAGTGTAGAG-3'	5'-ATCCAGTGCAGGGTCCGAGG-3'
miR-199a-3p	5'-GCGGCGGACAGTAGTCTGCAC-3'	5'-ATCCAGTGCAGGGTCCGAGG-3'
miR-199b-3p	5'-GCGGCGGACAGTAGTCTGCAC-3'	5'-ATCCAGTGCAGGGTCCGAGG-3'
miR-6509-3p	5'-GCGGCGGTTCCACTGCCACTAC-3'	5'-ATCCAGTGCAGGGTCCGAGG-3'
miR-9-5p	5'-GCGGCGGTCTTTGGTTATCTAG-3'	5'-ATCCAGTGCAGGGTCCGAGG-3'
miR-382-5p	5'-GCGGCGGGAAGTTGTTCGTGGTG-3'	5'-ATCCAGTGCAGGGTCCGAGG-3'
miR-526b-5p	5'-GCGGCGGCTCTTGAGGGAAGCAC-3'	5'-ATCCAGTGCAGGGTCCGAGG-3'
miR-432-5p	5'-GCGGCGGTCTTGGAGTAGGTC-3'	5'-ATCCAGTGCAGGGTCCGAGG-3'
miR-224-3p	5'-GCGGCGGAAAATGGTGCCCTAG-3'	5'-ATCCAGTGCAGGGTCCGAGG-3'
miR-522-3p	5'-GCGGCGGAAAATGGTTCCCTTTAG-3'	5'-ATCCAGTGCAGGGTCCGAGG-3'
miR-144-5p	5'-GCGGCGGGGATATCATCATATAC-3'	5'-ATCCAGTGCAGGGTCCGAGG-3'
U6	5'-CTCGCTTCGGCAGCACA-3'	5'-AACGCTTCACGAATTTGCGT-3'

Supplementary Table 3. ***AKT3*****-derived circRNAs according to the encyclopedia of RNA interactomes (ENCORI) and circinteractome databases.**

CircRNA ID	Position	Strand	Genomic length	Spliced length	ENCORI	CircInteractome
hsa_circ_0017242	chr1:243708811-244006584	-	297773	1363	√	√
hsa_circ_0112774	chr1:243708811-243801044	-	92233	822	×	√
hsa_circ_0017251	chr1:243776972-243859018	-	82046	650	√	√
hsa_circ_0112785	chr1:243772331-243772728	+	397	397	×	√
hsa_circ_0017249	chr1:243776972-243778463	-	1491	135	×	√
hsa_circ_0112773	chr1:243708811-243778463	-	69652	690	×	√
hsa_circ_0006696	chr1:243776972-243801044	-	24072	267	√	√
hsa_circ_0017252	chr1:243776972-244006584	-	229612	808	√	√
hsa_circ_0017243	chr1:243727021-243859018	-	131997	902	√	√
hsa_circ_0112797	chr1:243852874-243853025	+	151	151	×	√
hsa_circ_0112778	chr1:243723199-243723386	+	187	187	×	√
hsa_circ_0112800	chr1:243858892-243905345	-	46453	46453	×	√
hsa_circ_0004649	chr1:243858892-244006584	-	147692	284	√	√
hsa_circ_0112777	chr1:243716030-243801044	-	85014	734	×	√
hsa_circ_0017254	chr1:243809194-244006584	-	197390	541	√	√
hsa_circ_0017246	chr1:243736227-243828185	-	91958	647	√	√
hsa_circ_0112788	chr1:243788210-243791306	-	3096	3096	×	√
hsa_circ_0112798	chr1:243858892-243859018	-	126	126	×	√
hsa_circ_0017250	chr1:243776972-243828185	-	51213	524	√	√
**hsa_circ_0000199**	**chr1:243708811-243736350**	**-**	**27539**	**555**	√	√
hsa_circ_0112770	chr1:243675625-243727150	-	51525	535	×	√
hsa_circ_0112782	chr1:243727021-243809339	-	82318	664	×	√
hsa_circ_0112787	chr1:243778397-243859018	-	80621	581	×	√
hsa_circ_0017247	chr1:243736227-243859018	-	122791	773	√	√
hsa_circ_0017244	chr1:243727021-244006584	-	279563	1060	√	√
hsa_circ_0112767	chr1:243667689-243736350	-	68661	1605	×	√
hsa_circ_0112775	chr1:243716030-243736350	-	20320	467	×	√
hsa_circ_0017253	chr1:243800912-244006584	-	205672	673	√	√
hsa_circ_0112799	chr1:243858892-243899049	-	40157	40157	×	√
hsa_circ_0112776	chr1:243716030-243778463	-	62433	602	×	√
hsa_circ_0002240	chr1:243675625-243736350	-	60725	658	√	√
hsa_circ_0112801	chr1:243927962-243928163	-	201	201	×	√
hsa_circ_0112802	chr1:244006426-244006584	-	158	158	×	√
hsa_circ_0112772	chr1:243708811-243727689	-	18878	971	×	√
hsa_circ_0112780	chr1:243727021-243778463	-	51442	387	×	√
hsa_circ_0112771	chr1:243708811-243727150	-	18339	432	×	√
hsa_circ_0112791	chr1:243800912-243859018	-	58106	515	×	√
hsa_circ_0112766	chr1:243666011-243666150	-	139	139	×	√
hsa_circ_0112783	chr1:243736227-243778463	-	42236	258	×	√
hsa_circ_0017245	chr1:243736227-243777041	-	40814	192	√	√
hsa_circ_0112790	chr1:243800912-243828185	-	27273	389	×	√
hsa_circ_0112786	chr1:243776972-243809339	-	32367	412	×	√
hsa_circ_0112792	chr1:243800981-243828177	-	27196	312	×	√
hsa_circ_0112768	chr1:243668233-243668458	-	225	225	×	√
hsa_circ_0112784	chr1:243736227-243801044	-	64817	390	×	√

hsa_circ_0017248	chr1:243736227-244006584	-	270357	931	√	√
hsa_circ_0112779	chr1:243727021-243736350	-	9329	252	×	√
hsa_circ_0112796	chr1:243846498-243859018	-	12520	12520	×	√
hsa_circ_0112769	chr1:243673800-243674014	+	214	214	×	√
hsa_circ_0112789	chr1:243800912-243809233	-	8321	171	×	√
hsa_circ_0112781	chr1:243727021-243801044	-	74023	519	×	√
hsa_circ_0112794	chr1:243828073-243859018	-	30945	238	×	√
hsa_circ_0112793	chr1:243809194-243859018	-	49824	383	×	√
hsa_circ_0112795	chr1:243828073-243898418	-	70345	39638	×	√

*: ENCORI: https://www.starbase.sysu.edu.cn. CircInteractome: https://circinteractome.nia.nih.gov/index.html.

Supplementary Table 4. MiRNAs potentially targeted by *AKT3*-derived circRNAs in accordance with The Encyclopedia of RNA Interactomes (ENCORI) and CircInteractome databases*.

CircRNAs	Potentially targeted miRNAs*
hsa_circ_0017242	hsa-miR-613; hsa-miR-1-3p; hsa-miR-206; hsa-miR-147b; hsa-miR-450b-5p; hsa-miR-942-5p; hsa-miR-20a-5p; hsa-miR-106b-5p; hsa-miR-20b-5p; hsa-miR-526b-3p; hsa-miR-519d-3p; hsa-miR-93-5p; hsa-miR-17-5p; hsa-miR-106a-5p; hsa-miR-325; hsa-miR-382-3p; hsa-miR-337-3p; hsa-miR-656-3p; hsa-miR-3611; hsa-miR-516b-5p; hsa-miR-3129-5p; hsa-miR-199a-3p; hsa-miR-199b-3p; hsa-miR-6509-3p; hsa-miR-9-5p; hsa-miR-382-5p; hsa-miR-526b-5p; hsa-miR-432-5p; hsa-miR-224-3p; hsa-miR-522-3p; hsa-miR-144-5p
hsa_circ_0017251	hsa-miR-450b-5p; hsa-miR-942-5p; hsa-miR-20a-5p; hsa-miR-106b-5p; hsa-miR-20b-5p; hsa-miR-526b-3p; hsa-miR-519d-3p; hsa-miR-93-5p; hsa-miR-17-5p; hsa-miR-106a-5p; hsa-miR-325; hsa-miR-382-3p; hsa-miR-337-3p; hsa-miR-656-3p; hsa-miR-3611; hsa-miR-516b-5p; hsa-miR-3129-5p; hsa-miR-199a-3p; hsa-miR-199b-3p; hsa-miR-6509-3p; hsa-miR-9-5p; hsa-miR-382-5p; hsa-miR-526b-5p
hsa_circ_0006696	hsa-miR-450b-5p; hsa-miR-942-5p; hsa-miR-20a-5p; hsa-miR-106b-5p; hsa-miR-20b-5p; hsa-miR-526b-3p; hsa-miR-519d-3p;hsa-miR-93-5p; hsa-miR-17-5p; hsa-miR-106a-5p; hsa-miR-325; hsa-miR-382-3p
hsa_circ_0017252	hsa-miR-450b-5p; hsa-miR-942-5p; hsa-miR-20a-5p; hsa-miR-106b-5p; hsa-miR-20b-5p; hsa-miR-526b-3p; hsa-miR-519d-3p; hsa-miR-93-5p; hsa-miR-17-5p; hsa-miR-106a-5p; hsa-miR-325; hsa-miR-382-3p; hsa-miR-337-3p; hsa-miR-656-3p; hsa-miR-3611; hsa-miR-516b-5p; hsa-miR-3129-5p; hsa-miR-199a-3p; hsa-miR-199b-3p; hsa-miR-6509-3p; hsa-miR-9-5p; hsa-miR-382-5p; hsa-miR-526b-5p; hsa-miR-432-5p; hsa-miR-224-3p; hsa-miR-522-3p; hsa-miR-144-5p
hsa_circ_0017243	hsa-miR-613; hsa-miR-1-3p; hsa-miR-206; hsa-miR-147b; hsa-miR-450b-5p; hsa-miR-942-5p; hsa-miR-20a-5p; hsa-miR-106b-5p; hsa-miR-20b-5p; hsa-miR-526b-3p; hsa-miR-519d-3p; hsa-miR-93-5p; hsa-miR-17-5p; hsa-miR-106a-5p; hsa-miR-325; hsa-miR-382-3p; hsa-miR-337-3p; hsa-miR-656-3p; hsa-miR-3611; hsa-miR-516b-5p; hsa-miR-3129-5p; hsa-miR-199a-3p; hsa-miR-199b-3p; hsa-miR-6509-3p; hsa-miR-9-5p; hsa-miR-382-5p; hsa-miR-526b-5p
hsa_circ_0004649	hsa-miR-382-5p; hsa-miR-526b-5p; hsa-miR-432-5p; hsa-miR-224-3p; hsa-miR-522-3p; hsa-miR-144-5p
hsa_circ_0017254	hsa-miR-337-3p; hsa-miR-656-3p; hsa-miR-3611; hsa-miR-516b-5p; hsa-miR-3129-5p; hsa-miR-199a-3p; hsa-miR-199b-3p; hsa-miR-6509-3p; hsa-miR-9-5p; hsa-miR-382-5p; hsa-miR-526b-5p; hsa-miR-432-5p; hsa-miR-224-3p; hsa-miR-522-3p; hsa-miR-144-5p
hsa_circ_0017246	hsa-miR-613; hsa-miR-1-3p; hsa-miR-206; hsa-miR-147b; hsa-miR-450b-5p; hsa-miR-942-5p; hsa-miR-20a-5p; hsa-miR-106b-5p; hsa-miR-20b-5p; hsa-miR-526b-3p; hsa-miR-519d-3p; hsa-miR-93-5p; hsa-miR-17-5p; hsa-miR-106a-5p; hsa-miR-325; hsa-miR-382-3p; hsa-miR-337-3p; hsa-miR-656-3p; hsa-miR-3611; hsa-miR-516b-5p; hsa-miR-3129-5p; hsa-miR-199a-3p; hsa-miR-199b-3p; hsa-miR-6509-3p; hsa-miR-9-5p
hsa_circ_0017250	hsa-miR-450b-5p; hsa-miR-942-5p; hsa-miR-20a-5p; hsa-miR-106b-5p; hsa-miR-20b-5p; hsa-miR-526b-3p; hsa-miR-519d-3p; hsa-miR-93-5p; hsa-miR-17-5p; hsa-miR-106a-5p; hsa-miR-325; hsa-miR-382-3p; hsa-miR-337-3p; hsa-miR-656-3p hsa-miR-3611; hsa-miR-516b-5p; hsa-miR-3129-5p; hsa-miR-199a-3p; hsa-miR-199b-3p; hsa-miR-6509-3p; hsa-miR-9-5p

hsa_circ_0000199	hsa-miR-613; hsa-miR-1-3p; hsa-miR-206
hsa_circ_0017247	hsa-miR-613; hsa-miR-1-3p; hsa-miR-206; hsa-miR-147b; hsa-miR-450b-5p; hsa-miR-942-5p; hsa-miR-20a-5p; hsa-miR-106b-5p; hsa-miR-20b-5p; hsa-miR-526b-3p; hsa-miR-519d-3p; hsa-miR-93-5p; hsa-miR-17-5p; hsa-miR-106a-5p; hsa-miR-325; hsa-miR-382-3p; hsa-miR-337-3p; hsa-miR-656-3p; hsa-miR-3611; hsa-miR-516b-5p; hsa-miR-3129-5p; hsa-miR-199a-3p; hsa-miR-199b-3p; hsa-miR-6509-3p; hsa-miR-9-5p; hsa-miR-382-5p; hsa-miR-526b-5p
hsa_circ_0017244	hsa-miR-613; hsa-miR-1-3p; hsa-miR-206; hsa-miR-147b; hsa-miR-450b-5p; hsa-miR-942-5p; hsa-miR-20a-5p; hsa-miR-106b-5p; hsa-miR-20b-5p; hsa-miR-526b-3p; hsa-miR-519d-3p; hsa-miR-93-5p; hsa-miR-17-5p; hsa-miR-106a-5p; hsa-miR-325; hsa-miR-382-3p; hsa-miR-337-3p; hsa-miR-656-3p; hsa-miR-3611; hsa-miR-516b-5p; hsa-miR-3129-5p; hsa-miR-199a-3p; hsa-miR-199b-3p; hsa-miR-6509-3p; hsa-miR-9-5p; hsa-miR-382-5p; hsa-miR-526b-5p; hsa-miR-432-5p; hsa-miR-224-3p; hsa-miR-522-3p; hsa-miR-144-5p
hsa_circ_0017253	hsa-miR-450b-5p; hsa-miR-942-5p; hsa-miR-20a-5p; hsa-miR-106b-5p; hsa-miR-20b-5p; hsa-miR-526b-3p; hsa-miR-519d-3p; hsa-miR-93-5p; hsa-miR-17-5p; hsa-miR-106a-5p; hsa-miR-325; hsa-miR-382-3p; hsa-miR-337-3p; hsa-miR-656-3p; hsa-miR-3611; hsa-miR-516b-5p; hsa-miR-3129-5p; hsa-miR-199a-3p; hsa-miR-199b-3p; hsa-miR-6509-3p; hsa-miR-9-5p; hsa-miR-382-5p; hsa-miR-526b-5p; hsa-miR-432-5p; hsa-miR-224-3p; hsa-miR-522-3p; hsa-miR-144-5p
hsa_circ_0002240	hsa-miR-613; hsa-miR-1-3p; hsa-miR-206
hsa_circ_0017245	hsa-miR-613; hsa-miR-1-3p; hsa-miR-206; hsa-miR-147b
hsa_circ_0017248	hsa-miR-613; hsa-miR-1-3p; hsa-miR-206; hsa-miR-147b; hsa-miR-450b-5p; hsa-miR-942-5p; hsa-miR-20a-5p; hsa-miR-106b-5p; hsa-miR-20b-5p; hsa-miR-526b-3p; hsa-miR-519d-3p; hsa-miR-93-5p; hsa-miR-17-5p; hsa-miR-106a-5p; hsa-miR-325; hsa-miR-382-3p; hsa-miR-337-3p; hsa-miR-656-3p; hsa-miR-3611; hsa-miR-516b-5p; hsa-miR-3129-5p; hsa-miR-199a-3p; hsa-miR-199b-3p; hsa-miR-6509-3p; hsa-miR-9-5p; hsa-miR-382-5p; hsa-miR-526b-5p; hsa-miR-432-5p; hsa-miR-224-3p; hsa-miR-522-3p; hsa-miR-144-5p

*: ENCORI: https://www.starbase.sysu.edu.cn. CircInteractome: https://circinteractome.nia.nih.gov/index.html.

Supplementary Table 5. Correlation of miRNAs with autophagy-signalizing signaling pathways in triple-negative breast cancer.

miRNAs	Notch signaling	Wnt-βactin	PI3K/AKT/mTOR	EGFR protein	Triple-negative breast cancer
**hsa-miR-613**	[1]	[2]	[3]	×	[4]
hsa-miR-1-3p	[5]	×	[6]	[7]	[8]
**hsa-miR-206**	[9]	[10]	[11]	[12]	[13]
hsa-miR-147b	×	[14]	×	[15]	×
hsa-miR-450b-5p	×	[16]	×	×	×
hsa-miR-942-5p	×	[17]	×	×	×
hsa-miR-20a-5p	×	×	×	×	[18]
hsa-miR-106b-5p	[19]	[20]	×	×	×
hsa-miR-20b-5p	×	[21]	[22]	[23]	×
hsa-miR-526b-3p	×	×	×	×	×
hsa-miR-519d-3p	×	[24]	[25]	×	×
hsa-miR-93-5p	×	[26]	×	×	[27, 28]
hsa-miR-17-5p	[29]	[30]	[31]	[32]	×
hsa-miR-325	×	×	×	×	×
hsa-miR-382-3p	×	×	×	×	×
hsa-miR-337-3p	×	[33]	[33]	×	×
hsa-miR-656-3p	×	[20]	×	×	×
hsa-miR-3611	×	×	×	×	×
hsa-miR-516b-5p	×	[34]	×	×	×
hsa-miR-3129-5p	×	×	×	×	×
hsa-miR-199a-3p	[35]	[36]	[37]	[38]	[39]
hsa-miR-199b-3p	[40]	[41]	[42]	×	×
hsa-miR-6509-3p	×	×	×	×	×
hsa-miR-9-5p	[43]	[44]	×	×	[45]
hsa-miR-382-5p	×	×	×	×	×
hsa-miR-526b-5p	×	×	×	×	×
hsa-miR-432-5p	×	×	[46]	×	×
hsa-miR-224-3p	[47]	[48]	×	[49]	[50]
hsa-miR-522-3p	×	[51]	×	×	×
hsa-miR-144-5p	×	[52]	×	×	×

REFERENCES

1. Cai H, Yao J, An Y, Chen X, Chen W, Wu D, Luo B, Yang Y, Jiang Y, Sun D, He X. LncRNA HOTAIR acts a competing endogenous RNA to control the expression of notch3 via sponging miR-613 in pancreatic cancer. Oncotarget. 2017; 8:32905–17. https://doi.org/10.18632/oncotarget.16462 PMID:28415631

2. Anton R, Chatterjee SS, Simundza J, Cowin P, Dasgupta R. A systematic screen for micro-RNAs regulating the canonical Wnt pathway. PLoS One. 2011; 6:e26257. https://doi.org/10.1371/journal.pone.0026257 PMID:22043311

3. Li X, Sun X, Wu J, Li Z. MicroRNA-613 suppresses proliferation, migration and invasion of osteosarcoma by targeting c-MET. Am J Cancer Res. 2016; 6:2869–79. PMID:28042506

4. Wu J, Yuan P, Mao Q, Lu P, Xie T, Yang H, Wang C. miR-613 inhibits proliferation and invasion of breast cancer cell via VEGFA. Biochem Biophys Res Commun. 2016; 478:274–78. https://doi.org/10.1016/j.bbrc.2016.07.031 PMID:27449609

5. Furukawa S, Kawasaki Y, Miyamoto M, Hiyoshi M, Kitayama J, Akiyama T. The miR-1-NOTCH3-Asef pathway is important for colorectal tumor cell migration. PLoS One. 2013; 8:e80609. https://doi.org/10.1371/journal.pone.0080609 PMID:24244701

6. Wu J, Sun C, Wang R, Li J, Zhou M, Yan M, Xue X, Wang C. Cardioprotective effect of paeonol against epirubicin-induced heart injury via regulating miR-1 and PI3K/AKT pathway. Chem Biol Interact. 2018; 286:17–25. https://doi.org/10.1016/j.cbi.2018.02.035 PMID:29505745

7. Chiu KL, Lin YS, Kuo TT, Lo CC, Huang YK, Chang HF, Chuang EY, Lin CC, Cheng WC, Liu YN, Lai LC, Sher YP. ADAM9 enhances CDCP1 by inhibiting miR-1 through EGFR signaling activation in lung cancer metastasis. Oncotarget. 2017; 8:47365–78. https://doi.org/10.18632/oncotarget.17648 PMID:28537886

8. Jin C, Yan B, Lu Q, Lin Y, Ma L. Reciprocal regulation of Hsa-miR-1 and long noncoding RNA MALAT1 promotes triple-negative breast cancer development. Tumour Biol. 2016; 37:7383–94. https://doi.org/10.1007/s13277-015-4605-6 PMID:26676637

9. Wang XW, Xi XQ, Wu J, Wan YY, Hui HX, Cao XF. MicroRNA-206 attenuates tumor proliferation and migration involving the downregulation of NOTCH3 in colorectal cancer. Oncol Rep. 2015; 33:1402–10. https://doi.org/10.3892/or.2015.3731 PMID:25607234

10. Cui S, Li L, Mubarokah SN, Meech R. Wnt/β-catenin signaling induces the myomiRs miR-133b and miR-206 to suppress Pax7 and induce the myogenic differentiation program. J Cell Biochem. 2019; 120:12740–51. https://doi.org/10.1002/jcb.28542 PMID:30945349

11. Chen QY, Jiao DM, Wu YQ, Chen J, Wang J, Tang XL, Mou H, Hu HZ, Song J, Yan J, Wu LJ, Chen J, Wang Z. MiR-206 inhibits HGF-induced epithelial-mesenchymal transition and angiogenesis in non-small cell lung cancer via c-Met /PI3k/Akt/mTOR pathway. Oncotarget. 2016; 7:18247–61. https://doi.org/10.18632/oncotarget.7570 PMID:26919096

12. Choi BH, Ryu DY, Ryoo IG, Kwak MK. NFE2L2/NRF2 silencing-inducible miR-206 targets c-MET/EGFR and suppresses BCRP/ABCG2 in cancer cells. Oncotarget. 2017; 8:107188–205. https://doi.org/10.18632/oncotarget.22513 PMID:29291022

13. Wang J, Tsouko E, Jonsson P, Bergh J, Hartman J, Aydogdu E, Williams C. miR-206 inhibits cell migration through direct targeting of the actin-binding protein coronin 1C in triple-negative breast cancer. Mol Oncol. 2014; 8:1690–702. https://doi.org/10.1016/j.molonc.2014.07.006 PMID:25074552

14. Yue Y, Lv W, Zhang L, Kang W. MiR-147b influences vascular smooth muscle cell proliferation and migration via targeting YY1 and modulating Wnt/β-catenin activities. Acta Biochim Biophys Sin (Shanghai). 2018; 50:905–13. https://doi.org/10.1093/abbs/gmy086 PMID:30060075

15. Ning X, Wang C, Zhang M, Wang K. Ectopic expression of miR-147 inhibits stem cell marker and epithelial-mesenchymal transition (EMT)-related protein expression in colon cancer cells. Oncol Res. 2019; 27:399–406. https://doi.org/10.3727/096504018X15179675206495 PMID:29426374

16. Ye YP, Wu P, Gu CC, Deng DL, Jiao HL, Li TT, Wang SY, Wang YX, Xiao ZY, Wei WT, Chen YR, Qiu JF, Yang RW, et al. miR-450b-5p induced by oncogenic KRAS is required for colorectal cancer progression. Oncotarget. 2016; 7:61312–24. https://doi.org/10.18632/oncotarget.11016 PMID:27494869

17. Ge C, Wu S, Wang W, Liu Z, Zhang J, Wang Z, Li R, Zhang Z, Li Z, Dong S, Wang Y, Xue Y, Yang J, et al. miR-942 promotes cancer stem cell-like traits in esophageal squamous cell carcinoma through activation of Wnt/β-catenin signalling pathway. Oncotarget. 2015; 6:10964–77. https://doi.org/10.18632/oncotarget.3696 PMID:25844602

18. Bai X, Han G, Liu Y, Jiang H, He Q. MiRNA-20a-5p promotes the growth of triple-negative breast cancer cells through targeting RUNX3. Biomed Pharmacother. 2018; 103:1482–89. https://doi.org/10.1016/j.biopha.2018.04.165 PMID:29864933

19. Guarnieri AL, Towers CG, Drasin DJ, Oliphant MU, Andrysik Z, Hotz TJ, Vartuli RL, Linklater ES, Pandey A, Khanal S, Espinosa JM, Ford HL. The miR-106b-25 cluster mediates breast tumor initiation through activation of NOTCH1 via direct repression of NEDD4L. Oncogene. 2018; 37:3879–93. https://doi.org/10.1038/s41388-018-0239-7 PMID:29662198

20. Lu J, Wei JH, Feng ZH, Chen ZH, Wang YQ, Huang Y, Fang Y, Liang YP, Cen JJ, Pan YH, Liao B, Chen WF, Chen W, Luo JH. miR-106b-5p promotes renal cell carcinoma aggressiveness and stem-cell-like phenotype by activating Wnt/β-catenin signalling. Oncotarget. 2017; 8:21461–71. https://doi.org/10.18632/oncotarget.15591 PMID:28423523

21. Huang T, Alvarez AA, Pangeni RP, Horbinski CM, Lu S, Kim SH, James CD, J Raizer J, A Kessler J, Brenann CW,

Sulman EP, Finocchiaro G, Tan M, et al. A regulatory circuit of miR-125b/miR-20b and Wnt signalling controls glioblastoma phenotypes through FZD6-modulated pathways. Nat Commun. 2016; 7:12885. https://doi.org/10.1038/ncomms12885 PMID:27698350

22. He W, Cheng Y. Inhibition of miR-20 promotes proliferation and autophagy in articular chondrocytes by PI3K/AKT/mTOR signaling pathway. Biomed Pharmacother. 2018; 97:607–15. https://doi.org/10.1016/j.biopha.2017.10.152 PMID:29101804

23. Fu Q, Cheng J, Zhang J, Zhang Y, Chen X, Luo S, Xie J. miR-20b reduces 5-FU resistance by suppressing the ADAM9/EGFR signaling pathway in colon cancer. Oncol Rep. 2017; 37:123–30. https://doi.org/10.3892/or.2016.5259 PMID:27878272

24. Yue H, Tang B, Zhao Y, Niu Y, Yin P, Yang W, Zhang Z, Yu P. MIR-519d suppresses the gastric cancer epithelial-mesenchymal transition via Twist1 and inhibits Wnt/β-catenin signaling pathway. Am J Transl Res. 2017; 9:3654–64. PMID:28861156

25. Huang R, Lin JY, Chi YJ. MiR-519d reduces the 5-fluorouracil resistance in colorectal cancer cells by down-regulating the expression of CCND1. Eur Rev Med Pharmacol Sci. 2018; 22:2869–75. https://doi.org/10.26355/eurrev_201805_14989 PMID:29771440

26. Shi J, Jiang X, Yu Z, He G, Ning H, Wu Z, Cai Y, Yu H, Chen A. ZNRF3 contributes to the growth of lung carcinoma via inhibiting Wnt/β-catenin pathway and is regulated by miR-93. Tumour Biol. 2016; 37:3051–57. https://doi.org/10.1007/s13277-015-3949-2 PMID:26423400

27. Hu J, Xu J, Wu Y, Chen Q, Zheng W, Lu X, Zhou C, Jiao D. Identification of microRNA-93 as a functional dysregulated miRNA in triple-negative breast cancer. Tumour Biol. 2015; 36:251–58. https://doi.org/10.1007/s13277-014-2611-8 PMID:25238878

28. Shyamasundar S, Lim JP, Bay BH. miR-93 inhibits the invasive potential of triple-negative breast cancer cells in vitro via protein kinase WNK1. Int J Oncol. 2016; 49:2629–36. https://doi.org/10.3892/ijo.2016.3761 PMID:27840899

29. Zhang B, Chen M, Jiang N, Shi K, Qian R. A regulatory circuit of circ-MTO1/miR-17/QKI-5 inhibits the proliferation of lung adenocarcinoma. Cancer Biol Ther. 2019; 20:1127–35. https://doi.org/10.1080/15384047.2019.1598762 PMID:30975029

30. Liu W, Liu Y, Guo T, Hu C, Luo H, Zhang L, Shi S, Cai T, Ding Y, Jin Y. TCF3, a novel positive regulator of osteogenesis, plays a crucial role in miR-17 modulating the diverse effect of canonical Wnt signaling in different microenvironments. Cell Death Dis. 2013; 4:e539. https://doi.org/10.1038/cddis.2013.65 PMID:23492770

31. Luan Y, Chen M, Zhou L. MiR-17 targets PTEN and facilitates glial scar formation after spinal cord injuries via the PI3K/Akt/mTOR pathway. Brain Res Bull. 2017; 128:68–75. https://doi.org/10.1016/j.brainresbull.2016.09.017 PMID:27693649

32. Ohno M, Ohkuri T, Kosaka A, Tanahashi K, June CH, Natsume A, Okada H. Expression of miR-17-92 enhances anti-tumor activity of T-cells transduced with the anti-EGFRvIII chimeric antigen receptor in mice bearing human GBM xenografts. J Immunother Cancer. 2013; 1:21. https://doi.org/10.1186/2051-1426-1-21 PMID:24829757

33. Cui H, Song R, Wu J, Wang W, Chen X, Yin J. MicroRNA-337 regulates the PI3K/AKT and Wnt/β-catenin signaling pathways to inhibit hepatocellular carcinoma progression by targeting high-mobility group AT-hook 2. Am J Cancer Res. 2018; 8:405–21. PMID:29636997

34. Fang G, Ye BL, Hu BR, Ruan XJ, Shi YX. CircRNA_100290 promotes colorectal cancer progression through miR-516b-induced downregulation of FZD4 expression and Wnt/β-catenin signaling. Biochem Biophys Res Commun. 2018; 504:184–89. https://doi.org/10.1016/j.bbrc.2018.08.152 PMID:30173892

35. Ren K, Li T, Zhang W, Ren J, Li Z, Wu G. miR-199a-3p inhibits cell proliferation and induces apoptosis by targeting YAP1, suppressing Jagged1-notch signaling in human hepatocellular carcinoma. J Biomed Sci. 2016; 23:79. https://doi.org/10.1186/s12929-016-0295-7 PMID:27832779

36. Chen PH, Liu AJ, Ho KH, Chiu YT, Anne Lin ZH, Lee YT, Shih CM, Chen KC. microRNA-199a/b-5p enhance imatinib efficacy via repressing Wnt2 signaling-mediated protective autophagy in imatinib-resistant chronic myeloid leukemia cells. Chem Biol Interact. 2018; 291:144–51. https://doi.org/10.1016/j.cbi.2018.06.006 PMID:29890129

37. Fornari F, Milazzo M, Chieco P, Negrini M, Calin GA, Grazi GL, Pollutri D, Croce CM, Bolondi L, Gramantieri L. MiR-199a-3p regulates mTOR and c-met to influence

the doxorubicin sensitivity of human hepatocarcinoma cells. Cancer Res. 2010; 70:5184–93. https://doi.org/10.1158/0008-5472.CAN-10-0145 PMID:20501828

38. Liu R, Liu C, Zhang D, Liu B, Chen X, Rycaj K, Jeter C, Calhoun-Davis T, Li Y, Yang T, Wang J, Tang DG. miR-199a-3p targets stemness-related and mitogenic signaling pathways to suppress the expansion and tumorigenic capabilities of prostate cancer stem cells. Oncotarget. 2016; 7:56628–42. https://doi.org/10.18632/oncotarget.10652 PMID:27447749

39. Chen J, Shin VY, Siu MT, Ho JC, Cheuk I, Kwong A. miR-199a-5p confers tumor-suppressive role in triple-negative breast cancer. BMC Cancer. 2016; 16:887. https://doi.org/10.1186/s12885-016-2916-7 PMID:27842518

40. Liu MX, Siu MK, Liu SS, Yam JW, Ngan HY, Chan DW. Epigenetic silencing of microRNA-199b-5p is associated with acquired chemoresistance via activation of JAG1-Notch1 signaling in ovarian cancer. Oncotarget. 2014; 5:944–58. https://doi.org/10.18632/oncotarget.1458 PMID:24659709

41. Chen B, Zhang D, Kuai J, Cheng M, Fang X, Li G. Upregulation of miR-199a/b contributes to cisplatin resistance via Wnt/β-catenin-ABCG2 signaling pathway in ALDHA1^{+} colorectal cancer stem cells. Tumour Biol. 2017; 39:1010428317715155. https://doi.org/10.1177/1010428317715155 PMID:28639895

42. Torres A, Torres K, Pesci A, Ceccaroni M, Paszkowski T, Cassandrini P, Zamboni G, Maciejewski R. Deregulation of miR-100, miR-99a and miR-199b in tissues and plasma coexists with increased expression of mTOR kinase in endometrioid endometrial carcinoma. BMC Cancer. 2012; 12:369. https://doi.org/10.1186/1471-2407-12-369 PMID:22920721

43. Mohammadi-Yeganeh S, Mansouri A, Paryan M. Targeting of miR9/NOTCH1 interaction reduces metastatic behavior in triple-negative breast cancer. Chem Biol Drug Des. 2015; 86:1185–91. https://doi.org/10.1111/cbdd.12584 PMID:25963903

44. Yu T, Liu K, Wu Y, Fan J, Chen J, Li C, Yang Q, Wang Z. MicroRNA-9 inhibits the proliferation of oral squamous cell carcinoma cells by suppressing expression of CXCR4 via the Wnt/β-catenin signaling pathway. Oncogene. 2014; 33:5017–27. https://doi.org/10.1038/onc.2013.448 PMID:24141785

45. Jang MH, Kim HJ, Gwak JM, Chung YR, Park SY. Prognostic value of microRNA-9 and microRNA-155 expression in triple-negative breast cancer. Hum Pathol. 2017; 68:69–78. https://doi.org/10.1016/j.humpath.2017.08.026 PMID:28882698

46. Ma M, Wang X, Chen X, Cai R, Chen F, Dong W, Yang G, Pang W. MicroRNA-432 targeting E2F3 and P55PIK inhibits myogenesis through PI3K/AKT/mTOR signaling pathway. RNA Biol. 2017; 14:347–60. https://doi.org/10.1080/15476286.2017.1279786 PMID:28085550

47. Cong L, Zhang F, Shang H. Notch1 targeted regulation of mir-224/LRIG2 signaling for the proliferation and apoptosis of cervical cancer cells. Oncol Lett. 2017; 13:2304–08. https://doi.org/10.3892/ol.2017.5676 PMID:28454395

48. Liu F, Liu Y, Shen J, Zhang G, Han J. MicroRNA-224 inhibits proliferation and migration of breast cancer cells by down-regulating fizzled 5 expression. Oncotarget. 2016; 7:49130–42. https://doi.org/10.18632/oncotarget.9734 PMID:27323393

49. Chang ZW, Jia YX, Zhang WJ, Song LJ, Gao M, Li MJ, Zhao RH, Li J, Zhong YL, Sun QZ, Qin YR. LncRNA-TUSC7/miR-224 affected chemotherapy resistance of esophageal squamous cell carcinoma by competitively regulating DESC1. J Exp Clin Cancer Res. 2018; 37:56. https://doi.org/10.1186/s13046-018-0724-4 PMID:29530057

50. Zhang L, Zhang X, Wang X, He M, Qiao S. MicroRNA-224 promotes tumorigenesis through downregulation of caspase-9 in triple-negative breast cancer. Dis Markers. 2019; 2019:7378967. https://doi.org/10.1155/2019/7378967 PMID:30886656

51. Zhang H, Yu C, Chen M, Li Z, Tian S, Jiang J, Sun C. miR-522 contributes to cell proliferation of hepatocellular carcinoma by targeting DKK1 and SFRP2. Tumour Biol. 2016; 37:11321–29. https://doi.org/10.1007/s13277-016-4995-0 PMID:26960688

52. Guo Y, Ying L, Tian Y, Yang P, Zhu Y, Wang Z, Qiu F, Lin J. miR-144 downregulation increases bladder cancer cell proliferation by targeting EZH2 and regulating Wnt signaling. FEBS J. 2013; 280:4531–38. https://doi.org/10.1111/febs.12417 PMID:23815091
